# Establishment and characterization of a human juvenile bone marrow-derived mesenchymal stem/stromal cell line under advanced culture conditions for osteogenic differentiation

**DOI:** 10.3389/fbioe.2025.1719466

**Published:** 2026-01-07

**Authors:** Julia Moldaschl, Sofia Danilchenko, Isita Sagar, Vivian-Pascal Brandt, Heidrun Holland, Stephan Handschuh, Martin Glösmann, Stefan Toegel, Tobias May, Dominik Egger, Cornelia Kasper

**Affiliations:** 1 Institute of Cell and Tissue Culture Technologies, University of Natural Resources and Life Sciences, Vienna, Austria; 2 Saxonian Incubator for Clinical Translation (SIKT), University of Leipzig, Leipzig, Germany; 3 VetCore Facility for Research / Imaging Unit, University of Veterinary Medicine Vienna, Vienna, Austria; 4 Karl Chiari Lab for Orthopaedic Biology, Department of Orthopedics and Trauma Surgery, Medical University of Vienna, Vienna, Austria; 5 Ludwig Boltzmann Institute for Arthritis and Rehabilitation, Vienna, Austria; 6 InSCREENeX GmbH, Braunschweig, Germany; 7 Institute of Cell Biology and Biophysics, Leibniz University Hannover, Hanover, Germany

**Keywords:** 3D culture, cell line, juvenile, mesenchymal stem/stromal cell, osteogenic differentiation

## Abstract

Human mesenchymal stem/stromal cells (MSC) from juvenile donors (juvMSC) are crucial for studying bone development and for modeling pediatric skeletal diseases. However, the limited availability of these cells and the lack of physiologically relevant *in vitro* models hinder preclinical research. To address these issues, we established and characterized a new human bone marrow-derived MSC line under advanced culture conditions. Primary MSC from a 12-year-old donor in good health were immortalized via lentiviral transduction using a library of expansion genes. The resulting clone, C15 juvMSC, retained key features of MSC, including typical morphology, high proliferation rate, expression of stemness surface markers, and trilineage differentiation in a 3D format. Spectral karyotyping confirmed genomic stability without chromosomal aberrations. In 3D spheroid cultures, the C15 juvMSC demonstrated strong osteogenic potential, as evidenced by mineralization and alkaline phosphatase (ALP) activity. However, they exhibited a distinct differentiation pattern compared to primary cells. Overall, the C15 juvMSC line exhibits stable, scalable, and physiologically relevant characteristics, making it a valuable model for studying osteogenesis and for *in vitro* research on pediatric bone disorders.

## 1 Introduction

Mesenchymal stem/stromal cells (MSC) are widely recognized in regenerative medicine due to their self-renewal capacity, immunomodulatory properties, and multi-lineage differentiation potential (Chaudhary et al., 2018; Law and Chaudhuri, 2013). Consequently, they have emerged as a promising cell type for *in vitro* models. Specifically, their osteogenic differentiation capacity makes them an ideal tool for studying the molecular and cellular mechanisms of normal and pathological bone biology (Mitxitorena et al., 2019; Desiderio et al., 2014).

Compared to adult MSC, MSC derived from juvenile donors (juvMSC) have been shown to achieve higher cumulative population doublings (Mareschi et al., 2006) and exhibit elevated telomerase activity. Furthermore, juvMSC demonstrate enhanced differentiation capabilities, particularly for osteogenesis, reflecting their role in active skeletal growth and re-modeling during childhood (Knuth et al., 2018; Mueller and Glowacki, 2001; Siegel et al., 2013). These features confer a competitive advantage, as juvMSC more closely mirror the physiological conditions of active bone formation compared to MSC derived from adult donors, making them particularly suitable for studying the cellular and molecular mechanisms underlying osteogenesis.

Despite their high relevance, juvMSC are underrepresented in basic research. One possible explanation for this is their rarity. In fact, juvMSC are difficult to access since donor tissue is primarily obtained during infrequent clinical procedures, such as corrective orthopedic surgeries or bone marrow harvesting. This restricts both the frequency and volume of material that can be recovered (Nitkin and Bonfield, 2017). Furthermore, pediatric tissue procurement is subject to heightened ethical and legal oversight. This requires detailed parental informed consent and adherence to strict institutional review protocols, which constrains large-scale or routine collection. In addition to these challenges, primary MSC generally pose challenges to executing reproducible and comparable *in vitro* modeling (Piñeiro-Ramil et al., 2019). Specifically, primary MSC are limited by donor-to-donor variability (Phinney et al., 1999), a lifespan of ∼30–40 doublings, and inconsistency between batches (Heathman et al., 2016). These limitations are largely due to differences in donor characteristics, tissue sources, and processing methods (Viswanathan et al., 2014; Kabat et al., 2020). Furthermore, prolonged culture and passaging have been shown to reduce their utility by decreasing proliferation and differentiation potential, eventually inducing cellular senescence (Izadpanah et al., 2006; Izadpanah et al., 2008). Extended passaging has also been demonstrated to increase the risk of chromosomal instability, including clonal numerical and structural aberrations. Consequently, primary MSC from later passages are less potent and safe, and mechanistic studies based on them are not representative (Ross et al., 2011; Oliveira et al., 2014). To minimize donor-related variability, the present study employs an immortalized juvMSC line established from a single well-characterized donor. Such a cell line provides a stable, expandable, and genetically homogeneous population, thereby enabling long-term use and consistent experimentation in settings where large standardized cell numbers are required (Burk et al., 2019).

Most studies on MSC immortalization use the forced expression of human telomerase reverse transcriptase (hTERT), either alone or in combination with genes that interfere with the P53 and Rb-mediated pathways. Examples include the simian virus 40 large antigen (SV40 Tag) and the human papillomavirus E6/E7 genes (Wei et al., 2017; Tsai et al., 2010; L et al., 2013; Balducci et al., 2014; Takeda et al., 2004). Despite its benefits, hTERT immortalization can lead to increased immunogenicity and alter insulin- and cAMP-dependent signaling. These alterations can potentially impair the hormonal profile and adipogenic differentiation (Wolbank et al., 2009; Kulebyakin et al., 2021). The present study employed a previously described immortalization approach using lentiviral transduction with an expansion gene library, which enables efficient and stable genetic modification of primary cells. This technology has successfully immortalized various primary cell types, including osteoblasts and MSC isolated from adipose tissue and bone marrow derived from adult donors (Burk et al., 2019; Lipps et al., 2018).

In recent years, the scientific community has increasingly recognized the importance of replicating physiological conditions in MSC cell culture systems used for preclinical studies and *in vitro* modeling. While traditional culture methods have advanced bio-medical research, they often fail to accurately mimic the complex biochemical, mechanical, and structural environments of living tissues. This can limit the predictive power and reliability of data obtained from *in vitro* models, thus compromising the translational relevance of preclinical findings (Nikolits et al., 2021; McKee and Chaudhry, 2017). Consequently, increasing emphasis is being placed on the development of advanced culture systems that more accurately reflect human *in vivo* conditions. These systems incorporate factors such as hypoxic oxygen levels (Kwon et al., 2017; Zhao et al., 2020; Egger et al., 2024; Almeria et al., 2019), a culture medium that is free of xenogeneic serum and antibiotics (Palombella et al., 2022; Llobet et al., 2015), and three-dimensional (3D) spheroid cultures instead of traditional monolayer cultures (Egger et al., 2017; Oh et al., 2022; Yen et al., 2023). These improvements are particularly beneficial in the context of MSC culture. The formation of spheroids promotes stronger cell-to-cell communication, providing a setting that more accurately reflects their natural microenvironment. This results in improved stemness (Cheng et al., 2012), and anti-inflammatory properties (Bartosh et al., 2010), as well as more efficient differentiation into the adipogenic, chondrogenic and osteogenic lineage (Wang et al., 2009; Langenbach et al., 2011). Hypoxic conditions of 2%–5% O_2_, comparable to oxygen levels in the bone marrow niche, have been shown to increase MSC proliferation, delay senescence, and prime MSC for enhanced osteogenic differentiation (Kwon et al., 2017; Gu et al., 2016). Furthermore, the use of xenogeneic serum-free and aminoglycoside-free media minimizes variability and reduces unintended effects on MSC proliferation and differentiation, thereby supporting safer and more reproducible culture conditions (Palombella et al., 2022; Li and Yue, 2019; Skubis et al., 2017).

This study aimed to address the aforementioned critical questions to establish and characterize a human juvenile bone marrow-derived MSC line with robust osteogenic differentiation capacity. The C15 juvMSC line was generated by introducing a set of immortalizing transgenes into primary juvMSC. The cells were then selected and cultivated under hypoxic conditions using antibiotics- and xenogeneic serum-free media. The cell line was further compared to its primary counterparts and evaluated for its genetic background, cell morphology, proliferation potential, expression of stemness surface markers, and trilineage differentiation capacity in 3D spheroids.

## 2 Materials and methods

Unless otherwise stated, all reagents were purchased from Sigma-Aldrich, St. Louis, MO, United States.

### 2.1 MSC isolation and culture

MSC were isolated from 1 mL bone marrow aspirate obtained during temporary epiphysiodesis from a healthy 12-year-old male donor. The legal guardians of the donor gave written informed consent and the use of human bone marrow was approved by the ethics committee of the Medical University of Vienna (reference number 1158/2021). Following surgery, bone marrow aspirate was placed into cell culture flasks (TPP, Trasadingen, Switzerland) and maintained in an expansion medium composed of MEM alpha, 0.5% gentamicin, 2.5% fibrinogen-depleted human platelet lysate (hPL), and 1 U/mL heparin (the latter two were supplied by PL BioScience, Aachen, Germany). Cultures were incubated under humidified conditions at 37 °C, 5% CO_2_, and 21% O_2_. Once cell growth reached roughly 80% confluency, cells were detached with TrypLE (Thermo Fisher Scientific, Waltham, MA, United States) and preserved in cryomedium containing the aforementioned expansion medium, supplemented with 10% hPL (PL BioScience, Aachen, Germany) and 10% dimethyl sulfoxide, before storage in liquid nitrogen.

### 2.2 MSC immortalization and transduction

For lentiviral transduction of the gene library, the cryopreserved primary cells derived from juvenile MSC (primary juvMSC) were thawed and plated in expansion medium in passage two as described above. When the cells reached 80% confluency, they were transduced overnight with lentiviral vectors carrying a gene library consisting of 12 expansion genes, which represent a subset of a previously described library (Lipps et al., 2018). Transduction was performed at a multiplicity of infection (MoI) of five in the presence of polybrene (8 μg/mL). The following day, the virus-containing medium was removed, and cells were cultured for an additional 5 days before initiating selection with 0.2 mg/mL G418 (Thermo Fisher Scientific, Waltham, MA, United States). Selection continued for 14 days with medium replaced every 4 days, during which proliferating cell colonies became visible. These colonies were pooled, expanded without further selection, and subsequently used for downstream characterization.

### 2.3 Preliminary characterization and clone selection

The clone 15 (C15 juvMSC) was selected from a pool of nine independent cell lines cultured in antibiotic-free and xenogeneic serum-free culture medium composed of MEM alpha, 2.5% hPL, and 1 U/mL heparin under hypoxic conditions (37 °C, 5% CO_2_ and 5% O_2_). Primary juvMSC were used as a benchmark for the selection process, and the cell lines were evaluated based on the following criteria: (a) cell morphology, (b) proliferation rate, (c) cell size, (d) trilineage differentiation capacity in 3D, and (e) an MSC-specific immunophenotype (Supplementary Figures S1–S9).

### 2.4 Immunophenotyping

In accordance with the minimal criteria for identifying human multipotent MSC as outlined by the International Society for Cellular Therapy (ISCT; (Dominici et al., 2006)), MSC-specific surface antigen expression was assessed using the BD Stemflow™ Human MSC Analysis Kit (BD Biosciences, Franklin Lakes, NJ, United States), following the manufacturer’s protocol.

This kit detects the surface antigen markers CD73, CD90 and CD105, which must be expressed (≥95% positive), and the hematopoietic lineage markers CD34, CD45, CD11b, CD19 and HLA-DR, which must be absent (≤2% positive). Following staining, cells were resuspended in flow cytometry buffer (1% bovine serum albumin, 2 mM EDTA disodium salt dihydrate in phosphate-buffered saline (PBS, Gibco, Thermo Fisher Scientific, Waltham, MA, United States)). Data acquisition was performed on a CytoFLEX S4, recording 100,000 events per sample. Subsequent analysis was conducted using Kaluza Flow Cytometry Software, version 2.1 (both from Beckman Coulter, Brea, CA, United States).

### 2.5 Identification of integrated transgenes

To identify which expansion genes of the gene library were integrated in the selected C15 juvMSC clone (passage 14) in comparison with the parental primary cells (passage five), RNA was isolated from the cells, quantified with the NanoDrop lite Plus Spectrophotometer (Thermo Fisher Scientific, Waltham, MA, United States), and purified according to the manual of NucleoSpin RNA, Mini kit for RNA purification (MACHEREY-NAGEL GmbH & Co. KG, Düren, Germany). cDNA was synthesized by reverse transcription of 500 ng RNA following the manual of RevertAid RT Reverse Transcription Kit (Thermo Fisher Scientific, Waltham, MA, United States). qPCR was performed with the resulting cDNA in duplicates using TaqMan Fast Advanced PCR Kit on Quant StudioTM1 (Thermo Fisher Scientific, Waltham, MA, United States) and the associated software for the relative quantification of the Ct values of the target gene vs. the housekeeping gene POLR2A. The target genes, dye and primer sequences (Integrated DNA Technologies, Inc., Coralville, IA, United States) are shown in Table 1.

**TABLE 1:** Target genes, dye, and respective primer sequences.

Gene	Dye	Sequence forward	Sequence reverse	Sequence quencher
POLR2A	FAM	TCG​TCT​CTG​GGT​ATT​TGA​TGC	CAG​TTC​GGA​GTC​CTG​AGT​C	ACT​GAA​GCG​AAT​GTC​TGT​GAC​GGA​G
Bmi1	FAM	AAT​GCT​GGA​GAG​CTG​GAA​AG	ACT​GGA​AAT​GTG​AGG​GAA​CTG	TTC​CCT​CCA​CCT​CTT​CCT​GTT​TGC
Core	FAM	TTA​TGC​AAC​AGG​GAA​CCT​ACC	TCG​CGA​AGA​TCT​AGC​AGA​GA	TAT​CTT​CTT​GCT​GGC​CCT​GTT​GTC​C
E6	FAM	AAT​GTT​TCA​GGA​CCC​ACA​GG	GTT​GCT​TGC​AGT​ACA​CAC​ATT​C	AGC​GAC​CCA​GAA​AGT​TAC​CAC​AGT
E7	FAM	CGG​ACA​GAG​CCC​ATT​ACA​ATA	GAA​TGT​CTA​CGT​GTG​TGC​TTT​G	TGT​GAC​TCT​ACG​CTT​CGG​TTG​TGC
Fos	FAM	GGG​CAA​GGT​GGA​ACA​GTT​AT	CGC​TTG​GAG​TGT​ATC​AGT​CAG	TCC​GAA​GGG​AAA​GGA​ATA​AGA​TGG​CTG
ID1	FAM	GTG​CTG​CTC​TAC​GAC​ATG​AA	GAG​AAT​CTC​CAC​CTT​GCT​CAC	CCA​GCT​CCT​TGA​GGC​GTG​AGT​AAC
ID2	FAM	CAA​GAA​GGT​GAG​CAA​GAT​GGA	GGT​GAT​GCA​GGC​TGA​CAA​TA	TGC​AGC​ACG​TCA​TCG​ACT​ACA​TCT​TG
ID3	FAM	CGA​CAT​GAA​CCA​CTG​CTA​CTC	GAT​GAC​GCG​CTG​TAG​GAT​TT	AGT​CCC​GAG​AGG​CAC​TCA​GCT​TA
C-MYC	FAM	CTG​AGG​AGG​AAC​AAG​AAG​ATG​AG	TGT​GAG​GAG​GTT​TGC​TGT​G	AGA​GTC​TGG​ATC​ACC​TTC​TGC​TGG​A
Nanog	FAM	GGC​AGC​CCT​GAT​TCT​TCT​AC	GAG​AAC​ACA​GTC​CGC​ATC​TT	TGA​GGA​GGA​GGA​GAA​CAA​GGT​CCT
Rex	FAM	CAA​AGA​CAA​GTG​GCC​AGA​AAG	GGA​GCT​TCC​ACT​CTG​GTA​TTC	TGA​CCC​TAA​AGC​AAG​ACG​AGG​CAA
SV 40 Tag	FAM	GTG​GCA​TTG​CTT​TGC​TTC​TTA	GTC​CAA​TCT​CTC​TTT​CCA​CTC​C	AGA​CCT​GTG​GCT​GAG​TTT​GCT​CAA

### 2.6 Spectral karyotyping (SKY)

Karyotype analyses using spectral karyotyping (SKY) was performed on C15 juvMSC in passages 14 and 18, and primary juvMSC in passage four as a reference. The cells were incubated as described in Section 2.3. One cell culture flask (with a confluency of about 80%) per sample was used, respectively. The chromosome preparation was performed according to the protocol of Seabright et al. including the following steps: incubation with KaryoMAXTM Colcemid for 3 hours (Life Technologies GmbH, Darmstadt, Hesse, Germany, Art. No. 15210040), treatment with a hypotonic KCl solution followed by the rinse with a fixative solution as well as with methanol, and finally, the fixation of cells with fresh 3:1 methanol: acetic acid (Seabright, 1971). The cells were then applied onto pre-cleaned slides to allow chromosome spreading. Dried slides were applied for SKY analyses. These analyses were performed using SKYPAINT DNA H X10 (Applied Spectral Imaging GmbH, Edingen-Neckarhausen, Baden-Württemberg, Germany) according to the manufacturer’s instructions (Applied Spectral Imaging GmbH, Edingen-Neckarhausen, Baden-Württemberg, Germany). For the evaluation of numerical and structural chromosomal aberrations, karyotype analyses were performed.

### 2.7 Short tandem repeats (STR) profiling

Cell line authentication using short tandem repeat loci (STR) analyses was performed on C15 juvMSC in passage 15 (Microsynth AG, Balgach, Germany). STR loci were amplified using the PowerPlex® 16 HS System (Promega GmbH, Walldorf, Germany). Fragment analysis was done on an ABI3730xl (Life Technologies GmbH, Darmstadt, Germany) and the resulting data were analyzed with GeneMarker HID software (SoftGenetics, LLC, State College, PA, United States). Full analysis report is shown in Supplementary Figure S10.

### 2.8 Characterization of morphology and growth kinetics

For documentation of cell morphology and cell length, phase contrast microscopic images of primary juvMSC in passage two and of C15 juvMSC in passage 12 were obtained using a Leica DMi1. For cell size quantification of each cell type, 50 randomly selected cells were evaluated using FIJI (ImageJ, NIH, Bethesda, MD, United States). One linear measurement was taken for each cell along the longest and the shortest axes. These measurements were then averaged. To evaluate cell proliferation, the population doubling level (PDL) over eight consecutive passages was determined, corresponding to the point at which the primary cells entered the plateau phase and could not be further expanded. Primary juvMSC were analyzed from passages four to 11. In comparison, C15 juvMSC were examined from passages 14 to 21, with passage 21 reflecting the maximum passage tested in this study. The cells were plated at 2,500 cells/cm^2^ in four replicate wells and passaged by Accutase treatment every 72 h. The cumulative PDL was calculated using the following formula:

$$PDL=3.32*\left(\log\;\left[\text{cell count harvest}\right]-\log\;\left[\text{cell count seeding}\right]\right)+\text{previous PDL}$$

### 2.9 3D cell culture and trilineage differentiation

Primary juvMSC at passage four and C15 juvMSC at passage 14 were harvested using Accutase, and seeded into Sphericalplate 5D® low-attachment micro-patterned 24-well plates (Kugelmeiers, Erlenbach, Switzerland) at 500,000 cells per well, yielding approximately 670 cells per spheroid. After 24 h, once spheroid formation was observed, the culture medium described in Section 2.3 was replaced with the appropriate differentiation medium. Adipogenic differentiation was induced using MSCgo™ Adipogenic Differentiation Medium supplemented with MSCgo™ Adipogenic SF, XF Supplement Mix I and II; chondrogenic differentiation with MSCgo™ Chondrogenic Differentiation Medium supplemented with MSCgo™ Chondrogenic; and osteogenic differentiation with MSCgo™ Osteogenic Differentiation Medium. All differentiation media were free from xenogeneic compounds, serum, and antibiotics and were obtained from Sartorius, Göttingen, Germany. Differentiation proceeded under the aforementioned reduced oxygen conditions for up to 21 days, with medium changes every two to 3 days.

For all subsequent analyses, except high-resolution X-ray microscopy, spheroids were collected from at least three wells per cell type and time point.

#### 2.9.1 Differentiation-specific stainings

For all three differentiation lineages, spheroids were harvested after 21 days of differentiation. Spheroids harvested on day 0 served as a negative control.

Adipogenic differentiated spheroids were fixed with 4% paraformaldehyde for 1 hour at 4 °C, followed by permeabilization using 0.1% Triton-X for 30 min under continuous agitation. Samples were subsequently stained with Nile Red and DAPI, each at 5 μg/mL, to detect lipid droplets and nuclei, and then washed with PBS.

Chondrogenic spheroids were fixed in 4% paraformaldehyde for 1 hour and incubated overnight in 30% sucrose in PBS with agitation. Samples were embedded in Surgipath® FSC 22® Clear Frozen Section Compound (Biosystems Switzerland AG, Switzerland) and snap frozen. Sections of 7 µm thickness were cut using a Leica CM1950 cryostat. After thawing at room temperature, sections were rinsed twice for 5 minutes with PBS and incubated in 3% acetic acid for 5 minutes. Staining with 1% Alcian blue in 3% acetic acid (pH 2.5) was performed for 30 min to visualize sGAG in the extra-cellular matrix (ECM). Slides were rinsed under tap water for 9 minutes, counter-stained with 0.1% Nuclear Fast Red for 5 minutes, rinsed three times for 2 minutes with ddH_2_O, dehydrated through a graded alcohol series, mounted with Eukitt® medium, and coverslipped for imaging using a Leica DMi1 microscope.

For osteogenesis, spheroids were fixed in 70% ethanol for 30 min at 4 °C and subsequently stained overnight with calcein solution (5 μg/mL) under constant agitation to evaluate calcium phosphate deposition in the extracellular matrix. Nuclei were counterstained with DAPI (5 μg/mL) for 40 min, followed by six PBS washes. The imaging of adipogenic and osteogenic spheroids was performed using a Leica TCS SP8-STED laser scanning confocal microscope.

#### 2.9.2 Semi-quantitative image analysis

Processing of confocal microscopy images was performed as described in Section 2.8.1 and evaluated in FIJI (ImageJ) to quantify the abundance and distribution of differentiation-specific ECM molecules and nuclei signals. For adipogenesis, lipid droplets; for chondrogenesis, sGAGs; and for osteogenesis, calcium deposits were evaluated as lineage-specific ECM molecules. The ratio of characteristic ECM signal to nuclear signal was determined for adipogenic, chondrogenic, and osteogenic differentiation, while for the osteogenic lineage the area fraction of nuclear signal relative to the total spheroid area was additionally quantified, as previously described (Moldaschl et al., 2024). While an increase of the latter might reflect proliferation in the spheroid, a reduction in the nuclei-to-total spheroid area fraction may indicate mineral deposition in accordance with complementary analyses. For each sample, nine images were analyzed, and the mean values were calculated.

### 2.10 Osteogenic capacity

Osteogenic differentiated spheroids of primary juvMSC and C15 juvMSC harvested on day 21 (D21) and day 0 as negative control (D0) were subjected to the following analyses.

#### 2.10.1 Spheroid diameter analysis

Light microscopy images were captured with a Leica DMi1 at ×200 magnification. A minimum of ten images per sample type were analyzed using FIJI (ImageJ). Spheroid diameter was determined by measuring three angles per spheroid and calculating the average.

#### 2.10.2 Alkaline phosphatase (ALP) activity quantification

During osteogenic differentiation, culture medium was harvested on days 0, 1, 2, 3, 7, 10, 14, 17, and 21 and stored at −20 °C until analysis. Baseline readings from day 0 medium were used as blanks and subtracted from all subsequent measurements. Three wells per condition were analyzed, containing 2000 µL of cell culture medium, respectively. Cell culture medium was centrifuged for 5 minutes at 14,000 x g (4 °C) and 80 µL were transferred to the wells of a 96-well plate.

To each well, 20 µL of pNPP stock solution (prepared by dissolving one Tris buffer tablet and one SIGMAFAST™ p-nitrophenyl phosphate tablet in 4 mL ddH_2_O) was added, and the plate was incubated at 37 °C for 60 min. Absorbance at 405 nm was then recorded using an Infinite M1000 plate reader (Tecan, Männedorf, Switzerland). The amount of p-nitrophenolate generated was quantified against a calibration curve prepared from 4-nitrophenol in Tris buffer (one Tris tablet in 20 mL ddH_2_O). ALP activity U (µmol/min), defined as the enzyme amount converting 1 µmol of substrate per minute, was calculated using the following formula:

$$\text{ALP activity }U=\left(\frac{C_{\text{pNP}}}{0.1391}*t^{-1}\right)$$

C_pNP_ represents the concentration of p-nitrophenolate (µg/mL), 0.1391 is the conversion factor from µg/ml to µmol/L and t the incubation time (minutes). Volumetric enzyme activity was calculated as U × V, where V is the reaction volume (L).

### 2.11 High-resolution X-Ray microscopy

The spheroids were dehydrated in absolute ethanol and mounted in heat-sealed 10 µL micropipette tips. Each tip contained at least 50–100 spheroids in absolute ethanol, which sank to the bottom. A positive control sample consisting of grounded, decellularized and lyophilized human bone originating from femoral heads (Cells + Tissuebank Austria gemeinnützige GmbH, Allotec® process) was also prepared, dehydrated and mounted in a 10 µL micropipette tip. The top of each micropipette tip was sealed with several layers of parafilm to prevent evaporation. Each tip was then placed in a larger 200 µL pipette tip and fixed in the Xradia sample holder. Radiographs and microscopic X-ray computed tomography data were acquired using an XRadia MicroXCT-400 (Carl Zeiss X-ray Microscopy, Pleasanton, California, United States). The X-ray source settings were 40 kVp/200 μA, with no X-ray filter used. First, conventional radiographs were acquired using the 4X detector assembly, to check for mineralization in the day 21 spheroids that had undergone osteogenic differentiation. Next, an overview scan was acquired from the day 21 spheroids and the positive control sample using the 4X detector assembly (Supplementary Figure S11). The exposure time was set to 2 seconds per projection (detector binning = 1), with an angular increment of 0.33° between projections over a 360° rotation. The isotropic voxel size in the overview scan was 2.40 µm. Subsequently, high-resolution interior tomography was acquired from a selected region of interest in the day 21 spheroid samples using the 40x detector assembly, with an exposure time of 10 s per projection (detector binning = 2) and an angular increment of 0.225° between projections over a 360° rotation. Isotropic voxel size in the high-resolution scan was 0.48 µm. Image volumes were reconstructed using the XMReconstructor software supplied with the scanner and exported in *.TXM format. To image the non-mineralized parts of the spheroids as well, day 21 spheroids were dehydrated to absolute ethanol. Then, they were stained for 48 h in 0.5% (w/v) iodine solution in absolute ethanol (I_2_E) (Metscher, 2009), and mounted in 0.5% (w/v) I_2_E solution in 10-µL micropipette tips. Then, a scan was acquired with a 40X detector assembly at a voxel size of 0.48 µm. Tomographic image volumes were imported into the 3D reconstruction software Amira (FEI Visualization Sciences Group, part of Thermo Fisher Scientific, Mérignac Cédex, FR, version 2023.2). Prior to 3D visualization, the image volumes were filtered to reduce image noise. The forty-fold (40X) scans were processed with a 3D bilateral filter (kernel size: 3-3-3, similarity = 20,000) followed by a 3D Gaussian filter (kernel type: separable, kernel size factor = 2, standard deviation = 2-2-2). Image volumes were visualized using orthogonal slices and volume renderings.

#### 2.11.1 Mineral density measurements

Unstained primary juvMSC and C15 juvMSC spheroids on day 21 and the positive control were mounted in 10 µL micropipette tips and scanned using a µCT 35 (SCANCO Medical AG, Brüttisellen, CH) for quantitative measurements of mineral density. The µCT 35 includes a phantom-based calibration for bone mineral density measurements. The X-ray source settings were 70 kVp/114 μA, and the emitted X-ray spectrum was filtered by a 0.5-mm aluminum filter. The isotropic voxel size in the reconstructed volume was 3.5 µm. Tomograms were reconstructed using the scanner’s supplied software and exported in DCM format. Density-calibrated image volumes were imported into Amira software. The positive control sample was segmented using thresholding with a threshold of 400 mg HA/cm^3^. For each of the two spheroid samples, the highly mineralized core of ten spheroids was segmented manually. The periphery of C15 juvMSC day 21 spheroids was not visible in unstained samples because they showed almost no mineralization. In contrast, the periphery of primary juvMSC day 21 spheroids showed a larger amount of mineralization. However, due to the low spatial resolution of the datasets, the outer boundary of the spheroids could not be identified unambiguously. Thus, the periphery was also excluded from mineral density measurements (Supplementary Figure S12). Inside this manual segmentation mask, a threshold of 100 mg HA/cm^3^ was applied.

### 2.12 Statistical analysis

All results are presented as mean ± standard deviation. The sample size “n” for each experiment is indicated in the legend of the corresponding figure. Before performing statistical analyses, data normality was assessed using the Shapiro-Wilk test, followed by paired t-tests. Data visualization and statistical analyses were conducted using GraphPad Prism 8.0.0 for Windows (GraphPad Software, San Diego, CA, United States). Statistical significance is denoted as follows: *p < 0.05, **p < 0.01, and ***p < 0.001.

## 3 Results

As described in Section 2.3., nine independent clones were generated and screened to identify the most suitable cell line for in-depth characterization. Based on the outcomes of these preliminary experiments (Supplementary Figures S1–S9), the clone C15 juvMSC was selected and subjected to further studies as follows.

### 3.1 Evaluation of the integrated transgenes and the genetic integrity

**FIGURE 1:**
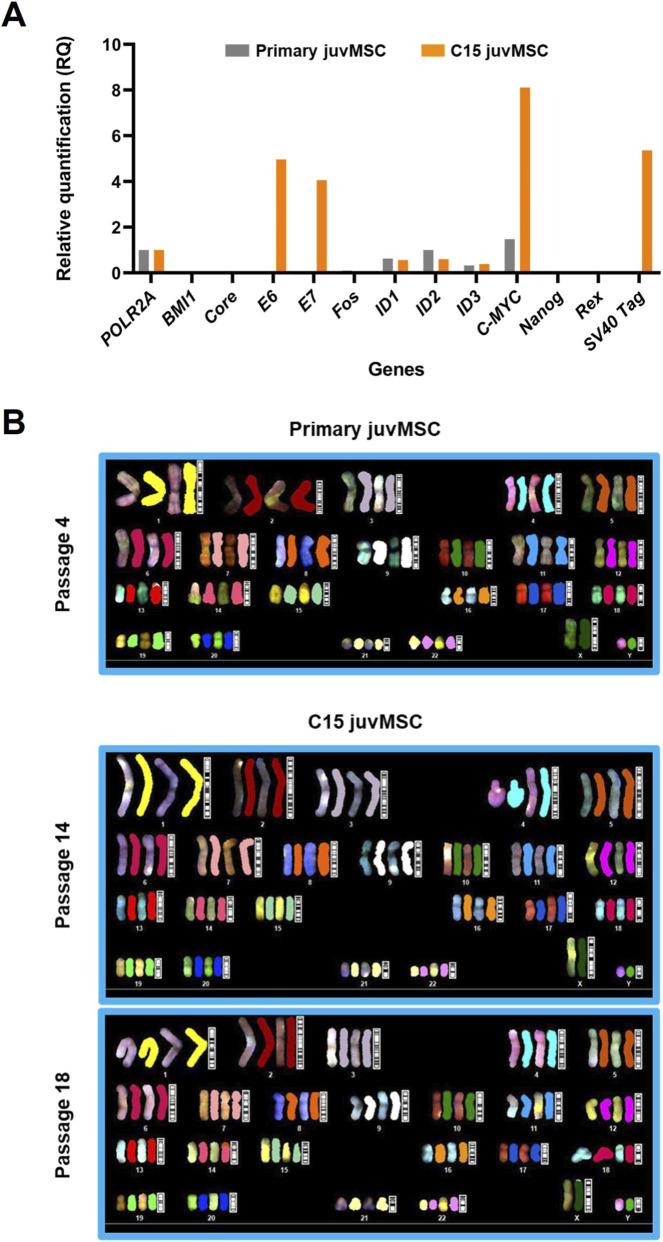
Integrated transgenes and genetic integrity. **(A)** qPCR analysis was performed to assess the expression of integrated transgenes from the expansion gene library. Target gene expression was evaluated in C15 juvMSC at passage 14 and compared to primary juvMSC at passage five as a reference. Expression levels were normalized to the housekeeping gene POLR2A and are presented as mean relative quantification (RQ) values. All qPCR reactions were performed in duplicate. **(B)** Spectral karyotyping (SKY) revealed no chromosomal aberrations. Chromosome analyses were performed on C15 juvMSC at passages 14 and 18, with primary juvMSC at passage four shown as a reference.

For immortalization, primary juvMSC were transduced with a gene library comprising BMI1, Core, E6, E7, Fos, ID1, ID2, ID3, c-MYC, Nanog, Rex, and SV40 Tag, as mentioned in Section 2.2. The qPCR analysis of the transgene integration pattern revealed the overexpression of four genes from this gene library in C15 juvMSC compared to the primary juvMSC: E6, E7, c-MYC, and SV40 Tag (Figure 1A). SKY analyses were applied to evaluate the genetic integrity of the parental primary juvMSC and C15 juvMSC at two distinct time points following immortalization. In all three samples, no indications for numerical or structural chromosomal aberrations were detected (Figure 1B).

### 3.2 Morphology, cell size, proliferation and immunophenotype

**FIGURE 2:**
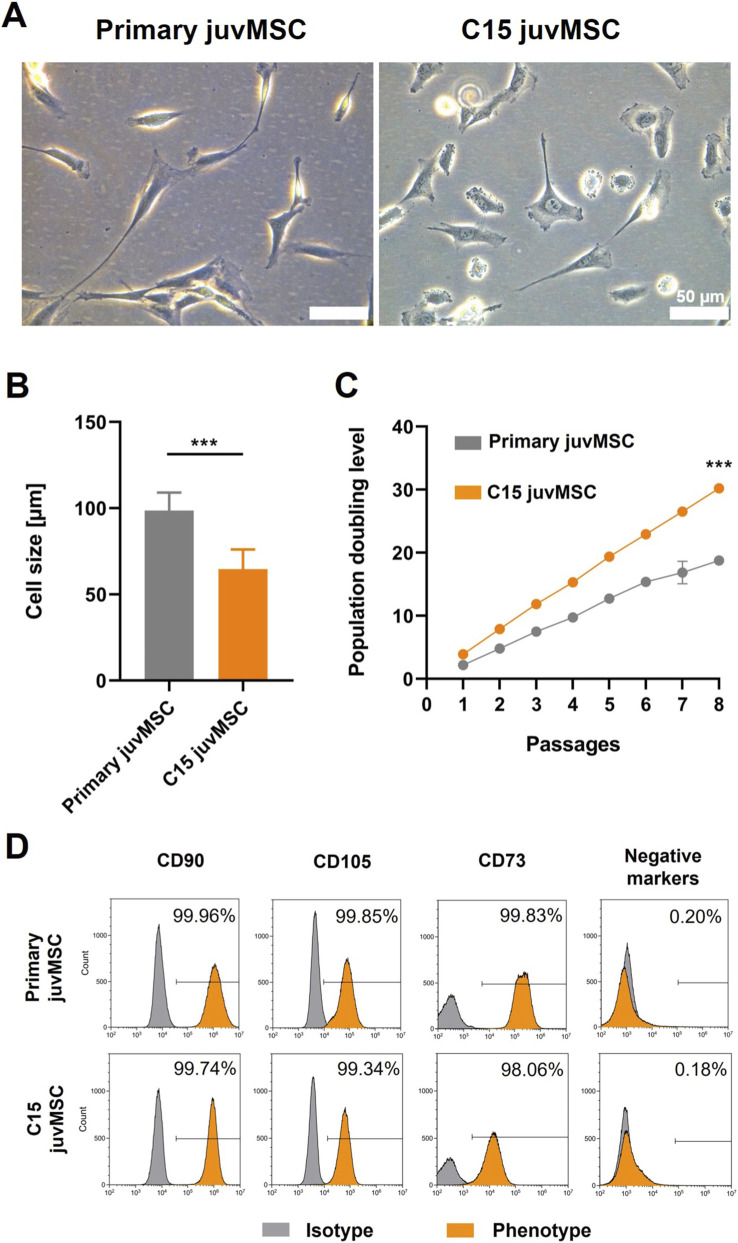
Cell culture characteristics. **(A)** Phase contrast micrographs showing the morphology of primary juvMSC and C15 juvMSC. **(B)** Quantification of cell size reveals that C15 juvMSC are significantly smaller. Bars show the mean of 50 randomly selected cells. **(C)** C15 juvMSC reached a higher population doubling level (PDL) over eight passages (n = 4 ± SD). Error bars indicate standard deviation. Normality of data was assessed using the Shapiro–Wilk test; statistical significance was determined by paired t-test (*p ≤ 0.05; **p ≤ 0.01; ***p ≤ 0.001). **(D)** Flow cytometric analysis confirmed that both primary and C15 juvMSC expressed MSC-related surface markers within the required range as proposed by the ISCT. Data represents 100,000 acquired events per sample.

Both primary and C15 juvMSC demonstrated plastic adherence, aligning with the MSC minimal criteria established by the ISCT (Dominici et al., 2006), with spindle-shaped, fibroblast-like morphologies. However, while primary juvMSC had elongated cell bodies with extended cellular processes, clone C15 juvMSC had a more polygonal or round shape with shorter protrusions (Figure 2A). Cell size measurements confirmed this observation (Figure 2B): C15 juvMSC had a significantly smaller average cell size (65 µm ± 11 µm) than primary juvMSC (98 µm ± 10 µm). Furthermore, C15 juvMSC demonstrated a significantly higher PDL over eight consecutive passages (PDL 30 ± 0.1) compared to primary juvMSC (PDL 19 ± 0.5), suggesting a more rapid proliferation pattern in the former (Figure 2C). Identical MSC-specific surface marker patterns were observed for both cell types, with ≥98.06% of cells expressing positive markers (C90, C105, and C73) and ≤0.20% of cells lacking negative marker expression (CD34, CD45, CD11b, CD19 and HLA-DR) (Figure 2D). These results suggest that both the primary juvMSC and clone C15 juvMSC meet the minimal set of cell surface marker criteria proposed by the ISCT (Dominici et al., 2006) for defining human MSC.

### 3.3 Trilineage differentiation potential in 3D

To assess the trilineage differentiation capacity in primary and C15 juvMSC spheroids, the characteristic ECM component accumulation for the adipogenic, chondrogenic and osteogenic lineage was evaluated. This determination was performed qualitatively after 0 and 21 days upon differentiation induction. For adipogenic differentiation, lipid droplets were visualized with Nile Red, while for chondrogenesis sGAG accumulation was verified with Alcian Blue staining. Calcein staining was used to assess calcium phosphate crystal deposition characteristic for osteogenic differentiation.

**FIGURE 3:**
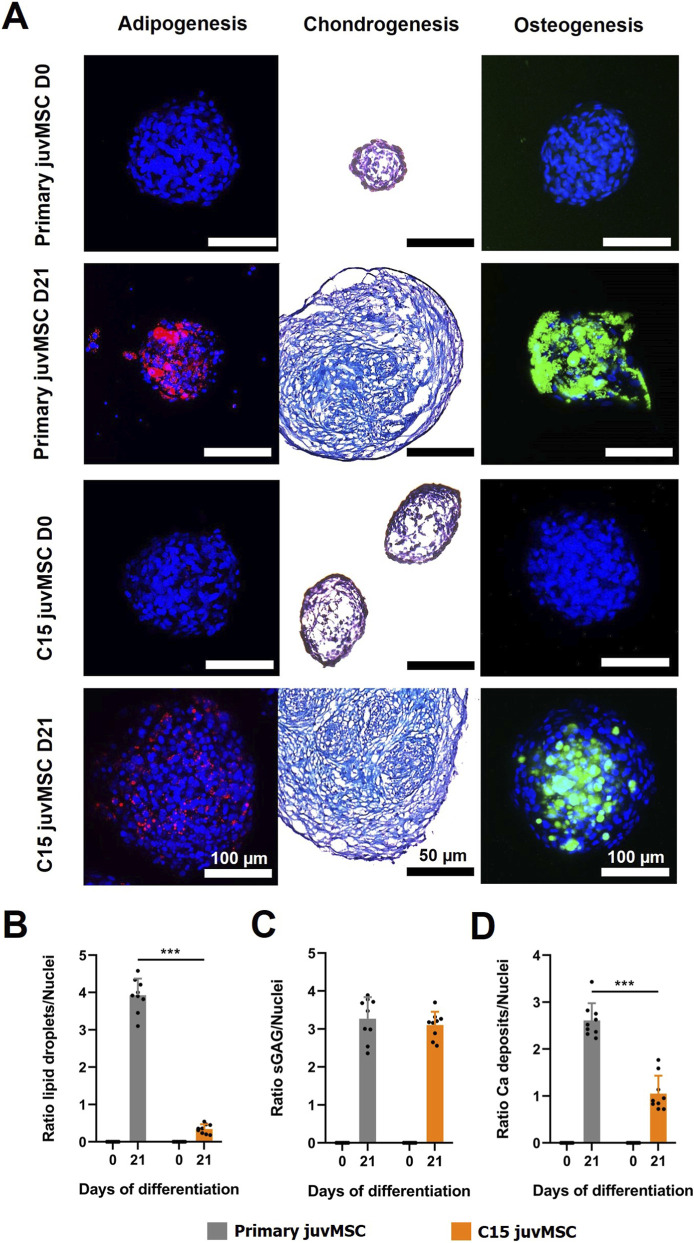
Trilineage differentiation capacity in 3D. **(A)** Representative images of primary and C15 juvMSC spheroids following 0 and 21 days of trilineage differentiation are shown. Following adipogenic differentiation, lipid droplets were stained with Nile Red (red), and nuclei were counterstained with DAPI (dark blue). For osteogenic differentiation, calcium phosphate crystals were labeled with Calcein (green), with DAPI used to stain nuclei. Fluorescence imaging of adipogenic and osteogenic spheroids was performed using scanning confocal microscopy. Chondrogenic spheroids were cryosectioned and stained with Alcian Blue for sulfated glycosaminoglycans (sGAG), while nuclei were counterstained with Nuclear Fast Red (purple). Light microscopy was used for imaging chondrogenic sections. Images shown are representative of nine images captured per condition. Semiquantitative analysis of the specific ECM staining is shown in **(B–D)** for adipogenic, chondrogenic and osteogenic differentiation, respectively. All values represent the mean of nine independently imaged and analyzed spheroids from at least three different wells for each cell type and time point, the error bars indicate the standard deviation. Data normality was assessed using the Shapiro–Wilk test; statistical significance was determined using a paired t-test (p ≤ 0.05; *p ≤ 0.01; **p ≤ 0.001).

This analysis revealed that C15 juvMSC accumulated lipid droplets, indicating adipogenic differentiation. However, after 21 days of differentiation the lipid vacuoles were smaller and the ratio of lipid droplets to nuclei significantly lower (0.4 ± 0.1) compared to those of primary juvMSC (3.9 ± 0.4) (Figures 3A,B). The presence of sGAG indicated a strong chondrogenic differentiation of C15 juvMSC, analogous to that observed in primary juvMSC (Figures 3A,C). In the context of osteogenesis (Figures 3A,D), the scanning confocal microscopy data demonstrated a substantial mineral deposition in C15 juvMSC, with crystals manifesting a round morphology, a predominantly homogeneous shape, and a preponderant location within the spheroid core. In contrast, calcium phosphate crystals in primary juvMSC spheroids exhibited variability in shape and size. These crystals were also observed in the peripheral region of the spheroids and were frequently arranged in plate-like structures. 3D rendering of the imaged spheroids can be seen in Supplementary Videos S13, S14. Despite the impaired adipogenic differentiation, clone C15 juvMSC demonstrated reliable trilineage differentiation in spheroids, thereby meeting the minimal criteria for defining human MSC as established by the ISCT (Dominici et al., 2006).

### 3.4 Osteogenesis in 3D

**FIGURE 4:**
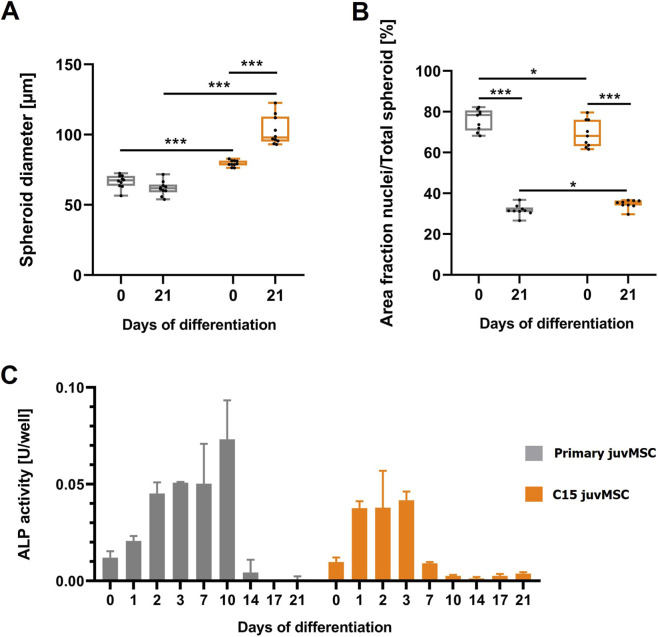
Osteogenic capacity in 3D. Spheroids derived from primary and C15 juvMSC were analyzed at defined time points to assess their osteogenic differentiation potential. **(A)** Spheroid diameter measured on days 0 and 21 of osteogenic induction. Semi-quantitative image analysis of the area fraction of nuclear signal **(B)** was performed at days 0 and 21. Microscope images were acquired and analyzed in ImageJ. **(C)** Alkaline phosphatase (ALP) activity, during osteo-genic differentiation of primary and C15 juvMSC spheroids. Data represent the mean of ten **(A)** or nine **(B)** independently imaged and analyzed spheroids. In **(C)**, values represent the mean of three independently measured wells. Error bars indicate standard deviation. Data normality was assessed using the Shapiro–Wilk test; statistical significance was determined using a paired t-test (p ≤ 0.05; *p ≤ 0.01; **p ≤ 0.001).

To more precisely characterize the osteogenic differentiation capacity of the proposed clone C15 juvMSC and its reference primary juvMSC, the following parameters were studied during the course of 21 days of osteogenic differentiation in spheroids: the spheroid size, the nuclei area fraction, the calcium phosphate crystals to nuclei area ratio, the secretion of the osteogenic-specific marker ALP, and the µ-CT analysis, examining the calcium phosphate mineralization pattern qualitatively and quantitatively, and thereby complementing the findings of the confocal microscopy analysis. The spheroid diameter (Figure 4A) of C15 juvMSC increased significantly after 21 days of osteogenesis (79 µm ± 2 μm–103 µm ± 10 µm), while primary juvMSC spheroids did not exhibit a significant change in size within the observed timespan (67 µm ± 4 μm–62 µm ± 5 µm). In general, the spheroids formed by C15 juvMSC exhibited a significant increase in size on both day 0 and day 21 of the differentiation process (day 0: 79 µm ± 2 μm, day 21: 103 µm ± 10 µm) compared to primary juvMSC (day 0: 67 µm ± 4 μm, day 21: 62 µm ± 5 µm).

The semi-quantitative analysis of the nuclei area fraction (Figure 4B) revealed a significant decline between days 0 and 21 of osteogenesis for both cell types. In detail, the nuclei share decreased from 76% ± 5% on day 0%–32% ± 3% on day 21 for primary juvMSC spheroids and from 69% ± 7%–35% ± 2% for the clone C15 juvMSC spheroid at the respective time points. The observed decrease in the nuclei area fraction during differentiation, when considered as a relative value in conjunction with an increase in spheroid size, can be indicative of calcium phosphate deposition and, consequently, successful osteogenic differentiation.

Figure 3D shows the results of the semi-quantitative image analysis of calcium phosphate to nuclei ratio. A significant increase in the ratio of primary juvMSC was observed after 21 days of osteogenic induction (2.6 ± 0.4) in comparison to C15 juvMSC (1.1 ± 0.4). However, both samples exhibited a marked increase in calcium phosphate deposition during the process of osteogenesis.

To assess the secretion of the early-osteogenesis marker ALP in the culture medium, the enzyme activity was quantitatively analyzed, with a particular emphasis on the initial week of differentiation (Figure 4C). For both primary juvMSC and C15 juvMSC, an increase in ALP activity was observed within the first days of osteogenesis. In the primary juvMSC sample, a peak was detected at day 10 with an ALP activity of 0.073 U/well, while C15 juvMSC peaked at day 3 with 0.042 U/well. Subsequent to the respective peak, the ALP activity decreased rapidly during further osteogenesis for both cell types. The integrated assessment of spheroid size, nuclei share, mineralization, and ALP secretion provides comprehensive insights into the osteogenic differentiation capacity of primary and C15 juvMSC.

### 3.5 Micro-CT analysis of osteogenic spheroids

**FIGURE 5:**
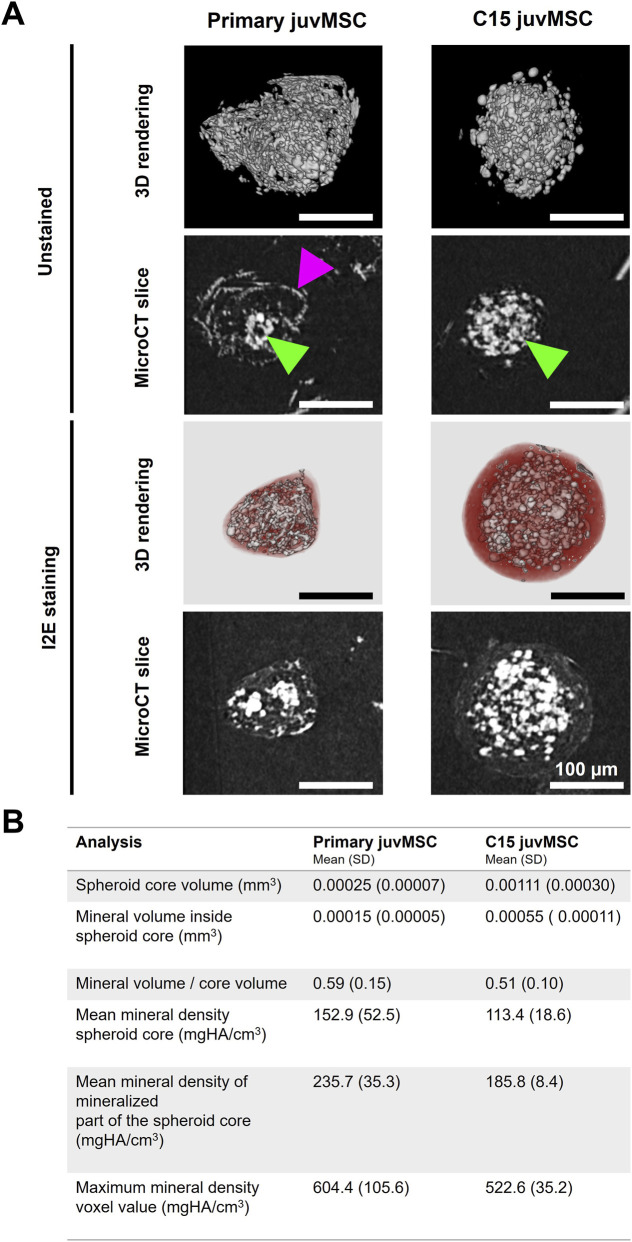
Micro-CT analysis of calcium phosphate crystals in primary juvMSC and C15 juvMSC spheroids after 21 days of osteogenic differentiation. **(A)** 3D rendering and micro-CT slices of unstained and I2E (iodine in ethanol) stained spheroids mounted and scanned in absolute ethanol. Green arrowheads = roundish calcium phosphate crystals in the spheroid core; magenta arrowheads = plate-like crystals in the spheroid periphery. **(B)** Quantitative bone mineral density measurements acquired using a µCT 35. All values represent the mean and standard deviation (SD) of ten measured spheroids.

The C15 juvMSC spheroids were nearly spherical, with mineralization restricted to the core. Mineralization was minimal in the peripheral regions. The calcium phosphate crystals in the spheroid cores were roundish. Primary juvMSC spheroids were irregularly shaped and varied in size. The spheroid cores were also found to contain round calcium phosphate crystals. Unlike C15 juvMSC spheroids, primary juvMSC spheroids exhibited mineralization in the periphery, where the crystals often had a plate-like morphology (Figure 5A).

#### 3.5.1 Mineral density measurements

In primary juvMSC spheroids, the mean volume of the mineralized core was 0.00025 mm^3^ (SD = 0.00007), and the mean mineral density of the entire core was 152.9 mg HA/cm^3^ (SD = 52.5). Setting a threshold of 100 mg HA/cm^3^ within the core yielded a mineral volume of 0.00015 (SD = 0.00005) and a mean mineral density of 235.7 mg HA/cm^3^ (SD = 35.5). The mean mineral volume-to-entire core volume ratio was found to be 0.59 (SD = 0.15), and the mean maximum mineral density voxel value was 604.4 mg HA/cm^3^ (SD = 105.6). A comparison of primary juvMSC spheroids and C15 juvMSC spheroids revealed that the latter had a larger core volume, but a lower mineral density. C15 juvMSC spheroids demonstrated a mean mineralized core volume of 0.00111 mm^3^ (SD = 0.00030) and a mean mineral density for the entire core of 113.4 mg HA/cm^3^ (SD = 18.6). Setting a threshold of 100 HA/cm^3^ inside the spheroid core yielded a mineral volume of 0.00055 mm^3^ (SD = 0.00011) and a mean mineral density of 185.8 mg HA/cm^3^ (SD = 8.4). The mean mineral volume-to-entire core volume ratio was 0.51 (SD = 0.10) and the mean maximum mineral density voxel value was determined to be 522.6 mg HA/cm^3^ (SD = 35.2) (Figure 5B; Supplementary Figure S15). The mean mineral density measured in the positive control was 730.94 mg HA/cm^3^ (SD = 179.9). These results complement the observations of the calcium phosphate crystal distribution and shape patterns depicted in Figure 3. Together with the observations in Figure 4, the data suggest that although osteogenesis is expressed in a distinct and reduced pattern compared to the primary counterpart, the spheroids derived from C15 juvMSC retain high osteogenic differentiation potential.

## 4 Discussion

This study established a human juvenile bone marrow-derived MSC line by lentiviral transduction of primary cells with an expansion gene library, based on a previously reported technique (Lipps et al., 2018). The generated C15 juvMSC line was characterized in detail, with an emphasis on its osteogenic differentiation capacity in 3D spheroids. To our knowledge, this is the first human juvenile MSC line cultured under multiple physiological conditions: serum- and antibiotic-free medium, low oxygen, and a spheroid format to study osteogenesis. Studies on the establishment of immortalized MSC derived from human pediatric donors with osteogenic differentiation potential are relatively rare and have not employed these advanced cell culture conditions (Wei et al., 2017; Song et al., 2017; Yadav et al., 2024).

The cell line development technique used in this study has been characterized as a rapid, efficient cell immortalization strategy applicable to a broad range of primary cells from various species, tissues, and donors (Lipps et al., 2018). Previous studies have shown that this technology successfully immortalizes human MSC derived from adipose tissue (Burk et al., 2019) and bone marrow (Lipps et al., 2018). In both cases, the cells were isolated from adult donors. The clone presented herein is the first human juvenile bone-marrow derived MSC line generated with this immortalization technique. This technology relies on random transduction using a lentiviral vector library encoding 12 genes associated with cell cycle progression, stemness maintenance, and senescence suppression (Takeda et al., 2004; Park et al., 2004; Bais et al., 2012; Bhandari et al., 2010). In the process of immortalizing the C15 juvMSC line, the oncogenes SV40 Tag, E6/E7, and c-MYC were integrated into the genome. c-MYC increases cell proliferation but reduces adipogenic, chondrogenic, and osteogenic differentiation. SV40 Tag and E6/E7 promote cell cycle progression by disrupting Rb- and p53-mediated pathways and can also influence osteogenic potential (Tsai et al., 2010; Melnik et al., 2019; Pieles et al., 2020). Although additional genes from the library, including Bmi1, Core, Fos, ID1, ID2, and ID3, Nanog and Rex were not detected in C15 juvMSC, these factors have been associated in other models with progenitor-state maintenance and the regulation of osteogenic and proliferative signaling (Takeda et al., 2004; Lipps et al., 2018; Park et al., 2004; Bais et al., 2012; Bhandari et al., 2010; Abarrategi et al., 2018; Roschger and Cabrele, 2017; Lasorella et al., 2001). Consistent with this variability, other MSC types immortalized using the same technology have exhibited distinct transgene integration patterns. Burk et al. (2019) reported that a total of 12 genes from the gene library were detected in the adipose tissue-derived MSC line. These genes include E7 and SV40 Tag, but not E6 and c-MYC. Lipps et al. (2018) found that six to 12 genes were integrated into the genome in five generated adult bone-marrow derived MSC lines, comprising all transgenes detected in C15 juvMSC except for SV40 Tag. These findings align with prior studies, suggesting that the ideal gene subset for promoting functional cell expansion depends on factors such as cell type, age, and donor specific characteristics.

It is important to acknowledge that long-term culture of immortalized cells can, in rare cases, lead to reduced proliferative capacity or decreased differentiation potential, particularly at very late passages (Yamaguchi et al., 2024). These risks are not unique to C15 juvMSC but apply broadly to immortalized cell lines, underscoring the importance of continuous monitoring and functional validation during extended culture. Long-term, expandable cell cultures are nonetheless essential for ensuring experimental reproducibility and achieving the high cell numbers necessary for robust *in vitro* models. In this context, the C15 juvMSC clone demonstrated superior and sustained proliferative capacity compared to its primary juvMSC counterpart. This finding is consistent with previous results obtained with the same transgenes in adult bone marrow-derived MSC (Lipps et al., 2018) and aligns with the proliferation pattern reported for other juvenile MSC lines (Wei et al., 2017; Song et al., 2017; Yadav et al., 2024). Rigorous validation of phenotypic stability and functionality is necessary when establishing cell lines for use in *in vitro* modeling to closely recapitulate the properties of primary cells. Therefore, corresponding primary juvenile MSC were used as the reference standard throughout all phases of clonal evaluation to ensure direct comparison between the immortalized line and its juvenile counterparts. Key criteria for maintaining the primary cell phenotype included adherence to plastic surfaces, high expression of positive surface markers, absence of negative hematopoietic surface markers, and trilineage differentiation potential (Dominici et al., 2006), validated in the spheroid format. The C15 juvMSC line’s confirmed trilineage differentiation capacity contrasts with that of previously described immortalized juvenile MSC models, for which the potential for chondrogenesis has not been detected (Song et al., 2017) or tested (Wei et al., 2017; Sugimoto et al., 2017). However, adipogenesis was significantly reduced compared with primary juvMSC. This reduction likely reflects a combination of developmental and immortalization-related factors. MSC from fetal and perinatal sources naturally favor osteogenic over adipogenic differentiation, and the immortalization process may render C15 juvMSC functionally ‘younger’ than the primary cells (Z et al., 2009; Ragni et al., 2013). Additionally, the integration of c-MYC, known to suppress adipogenic differentiation, likely further contributes to the diminished adipogenic response (Melnik et al., 2019).

The C15 juvMSC clone retained the spindle-shaped morphology characteristic of primary juvMSC. However, the cells exhibited a more rounded appearance and were smaller in size. This observation is consistent with a previous report that employed similar immortalization strategies (Burk et al., 2019). Notably, these morphological features may indicate the selection of a MSC subpopulation with enhanced stemness properties (Bal et al., 2012), and increased proliferative and differentiation potential (Smith et al., 2004).

The C15 juvMSC line exhibited distinct characteristics of osteogenic differentiation compared to its primary MSC counterpart. After 21 days of osteogenic induction, the spheroids of the cell line were larger but had a lower calcium-to-nuclei ratio. Mineral density measurements based on micro-CT analysis supported this finding, revealing a greater spheroid core volume but reduced mineral density in the clone. This suggests that mineralization is slightly diminished, but still strong. Beyond differences in mineral content, notable distinctions were observed in the distribution and morphology of calcium phosphate crystals. C15 juvMSC formed mostly round crystals in the spheroid core, while primary juvMSC showed a more even crystal distribution, with plate-like structures at the periphery. Furthermore, ALP activity, an early marker of osteogenic differentiation, peaked earlier and was generally lower in magnitude in C15 juvMSC compared to primary cells. This phenomenon may underlie the lower calcium phosphate deposition observed in the cell line. Taken together, the data suggest that the C15 juvMSC cell line has a slightly reduced, yet highly efficient and robust, osteogenic differentiation capacity. Similar reductions in osteogenic capacity coupled with enhanced proliferative capacity have been reported in other SV40 large T antigen-immortalized MSC lines (Balducci et al., 2014; Tátrai et al., 2012). Nevertheless, the osteogenic potential of C15 juvMSC requires comprehensive characterization, which will necessitate future whole-genome sequencing to assess potential alterations in key differentiation-related genes.

Given that genetic instability poses a significant challenge during long-term cell expansion (Vukicevic et al., 2010), SKY analysis was performed. This analysis revealed no numerical or structural chromosomal aberrations attributable to the immortalization process or subsequent *in vitro* cultivation. Although SKY has the advantage of analyzing all chromosomes simultaneously in individual metaphase spreads and enabling the detection of cryptic interchromosomal rearrangements, it has inherent limitations in identifying intrachromosomal alterations. Specifically, small-scale chromosomal aberrations that fall below the resolution threshold of light microscopy cannot be detected using this method (Fan et al., 2000). Beyond this technical limitation, it is important to note that the C15 juvMSC line is not intended for clinical application or use in cell transplantation therapies. This is due to the continued risk of malignant transformation associated with MSC immortalization; the transgenes inserted into the C15 juvMSC genome are connected to oncogenic pathways (Moriya et al., 1998; Brinster et al., 1984). Importantly, further molecular profiling represents a valuable future step to complement the present characterization. While SKY analysis confirmed chromosomal stability, STR profiling established a unique genetic fingerprint for reliable authentication across laboratories, and functional benchmarking demonstrated preservation of key MSC properties, additional analyses, such as whole-genome or whole-exome sequencing and insertion site mapping, would further strengthen the genetic characterization of the line.

The C15 juvMSC cell line was established and characterized under defined, physiologically relevant culture conditions (hypoxia and xenogeneic, serum- and antibiotic-free media), which were deliberately selected to more closely mimic the native stem cell niche. This enhances the physiological relevance and translational potential of the model. The osteogenic and trilineage differentiation capacities of the C15 juvMSC were evaluated in spheroids grown in micropatterned multiwell plates, based on a previously described 3D MSC differentiation system (Moldaschl et al., 2024). This scaffold-free, 3D format is technically straightforward and scalable. It supports the reproducible generation of spheroids and renders this cell line system highly suitable for long-term *in vitro* studies and systematic condition testing. An important limitation to consider is that the immortalization strategy used to establish C15 juvMSC relied on the forced expression of oncogenes that can influence differentiation processes, as mentioned above. Our functional benchmarking indicated that osteogenic and trilineage differentiation capacities were preserved, yet the possibility of subtle intracellular influences cannot be entirely excluded. This highlights the inherent limitations of immortalized models. These cells are useful for developing complex protocols, generating preliminary data, and studying mechanisms, but they cannot replace primary MSC when donor-specific variability or clinical applications are important. Accordingly, C15 juvMSC, like immortalized and commercially available cell lines in general, should be regarded as a complementary tool to primary MSC, offering reproducibility and scalability while still requiring validation of key mechanistic findings in primary cells.

## 5 Conclusion

This study introduces the novel human bone marrow-derived MSC line C15 juvMSC, derived from a juvenile, healthy donor and immortalized by lentiviral transduction with c-MYC, E6, E7 and SV40. The cell line was established under advanced, defined culture conditions and was fully characterized. Notably, its core mesenchymal properties closely resemble those of the corresponding primary juvenile MSC. Proliferative potential was enhanced, and genetic integrity was maintained following immortalization and extended cultivation. Despite minor differences relative to the native primary cells, C15 juvMSC demonstrated robust osteogenic differentiation capacity in 3D spheroids, as assessed by ALP activity and matrix mineralization. Taken together, these features underscore the value of C15 juvMSC as a versatile, reliable and physiologically highly relevant resource for advancing fundamental research and broader preclinical studies. In its native form, the cell line is a promising candidate for standardized studies of osteogenesis and bone development. With further genetic or pharmacological modifications, it can be adapted to model pediatric skeletal diseases such as osteosarcoma, Ewing sarcoma, osteogenesis imperfecta, and hypophosphatasia. To support reproducibility and broader use, the C15 juvMSC cell line will be made available to the research community through collaborative agreements governed by material transfer agreements (MTAs).

## Data Availability

The raw data supporting the conclusions of this article will be made available by the authors, without undue reservation.
