# Supplementary material for: Establishment and characterization of a human juvenile bone marrow-derived mesenchymal stem/stromal cell line under advanced culture conditions for osteogenic differentiation
Source: Front Bioeng Biotechnol. 2026 Jan 7;13:1719466. doi: 10.3389/fbioe.2025.1719466 (PMC12819625; doi:10.3389/fbioe.2025.1719466)
Supplement: Supplementary file 2 [file DataSheet1.pdf]

Supplementary Material

1 Preliminary characterization and clone selection

The C15 juvMSC clone was selected from a pool of nine independently generated cell lines, using primary juvMSC as the reference standard (Figure S1). The evaluation followed a two-step process: Selection 1 assessed cell morphology (Figure S2) and proliferation rate (Figures S3, S4), while Selection 2 focused on cell size (Figure S5), trilineage differentiation capacity in 3D (Figure S6-8), and MSC-specific immunophenotype (Figure S9). In the first step, clones 3, 6, and 15 were shortlisted for further analysis. During the second step, C15 emerged as the most suitable candidate for in-depth characterization. Compared to the other clones, C15 most closely resembled the primary cells in morphology, proliferation, and cell size, while also exhibiting robust trilineage differentiation, the highest calcium deposit–to–nucleus ratio, and a characteristic MSC surface marker profile.

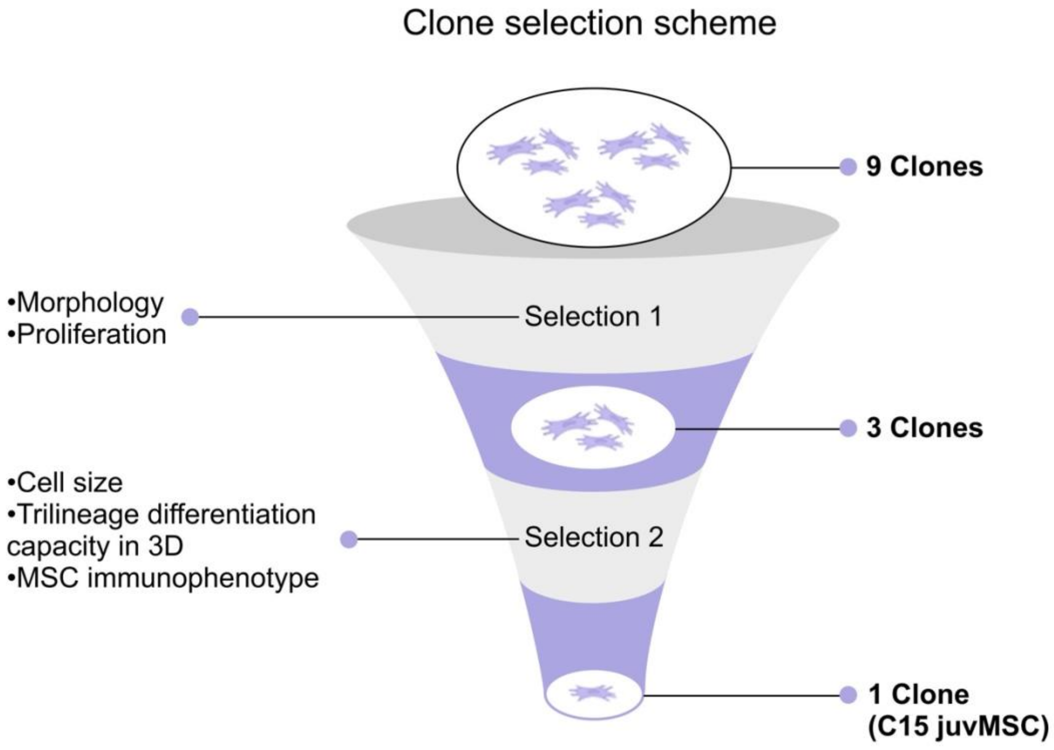

Supplementary Figure 1. Clone selection scheme.

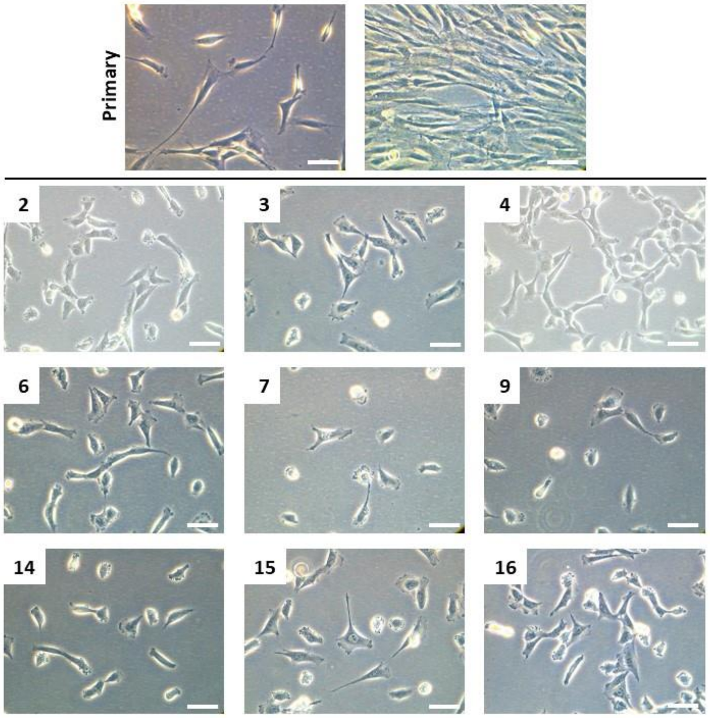

Supplementary Figure 2. Phase contrast microphotographs. Representative images of primary juvMSC and nine juvMSC clonal cell lines. Despite some divergence from the primary MSC phenotype, clones 3, 4, 6, 15, and 16 displayed features reminiscent of primary MSC. Notably, clones 6 and 15 exhibited elongated morphologies with prominent protrusions. All images were captured at 20X magnification. The scale bar, visible on each image, corresponds to 50 µm.

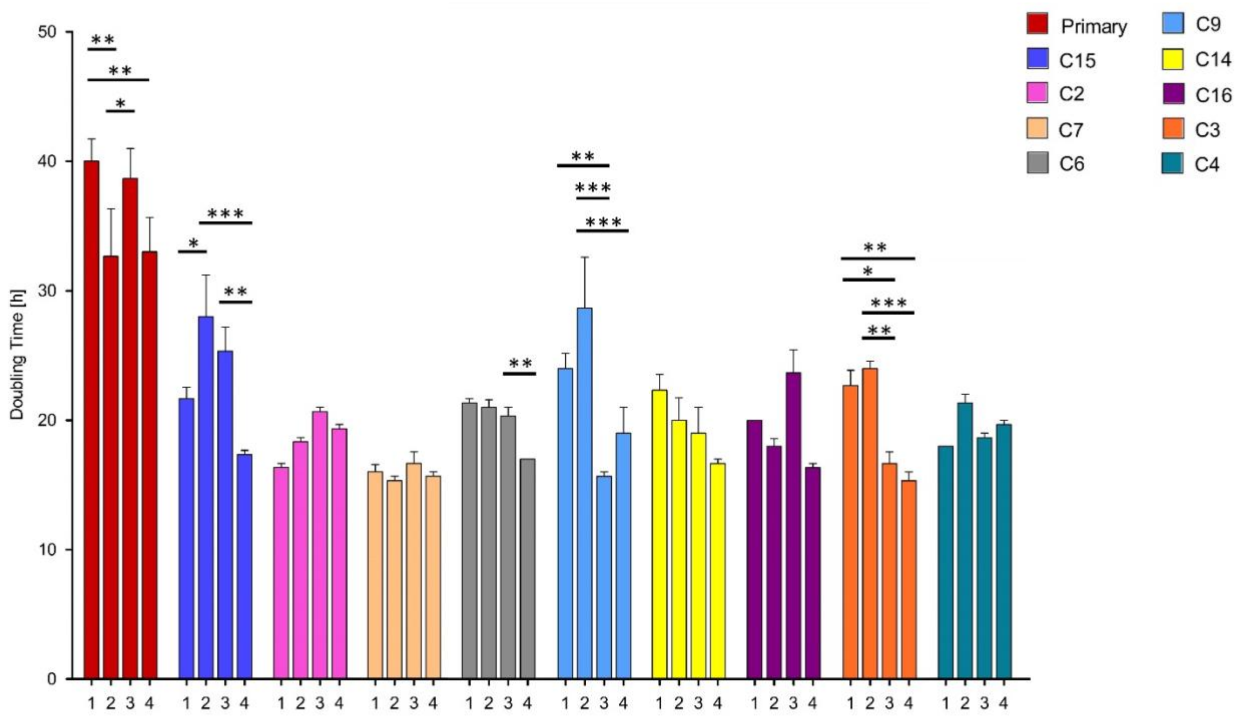

Supplementary Figure 3. Doubling time over four consecutive passages. Measurements were performed every 72 hours for primary juvMSC and nine juvMSC clones. Clones (passages 14 to 18) displayed overall stable proliferation dynamics and kinetics comparable to primary MSC (passages 4 to 8). For statistical analysis, a two-way ANOVA followed by Bonferroni post-test was used to compare DT values between groups. Bars represent the mean DT (n = 3), error bars denote standard deviation, and statistical significance is indicated as follows: *p < 0.05, **p < 0.01, ***p < 0.001, ****p < 0.0001.

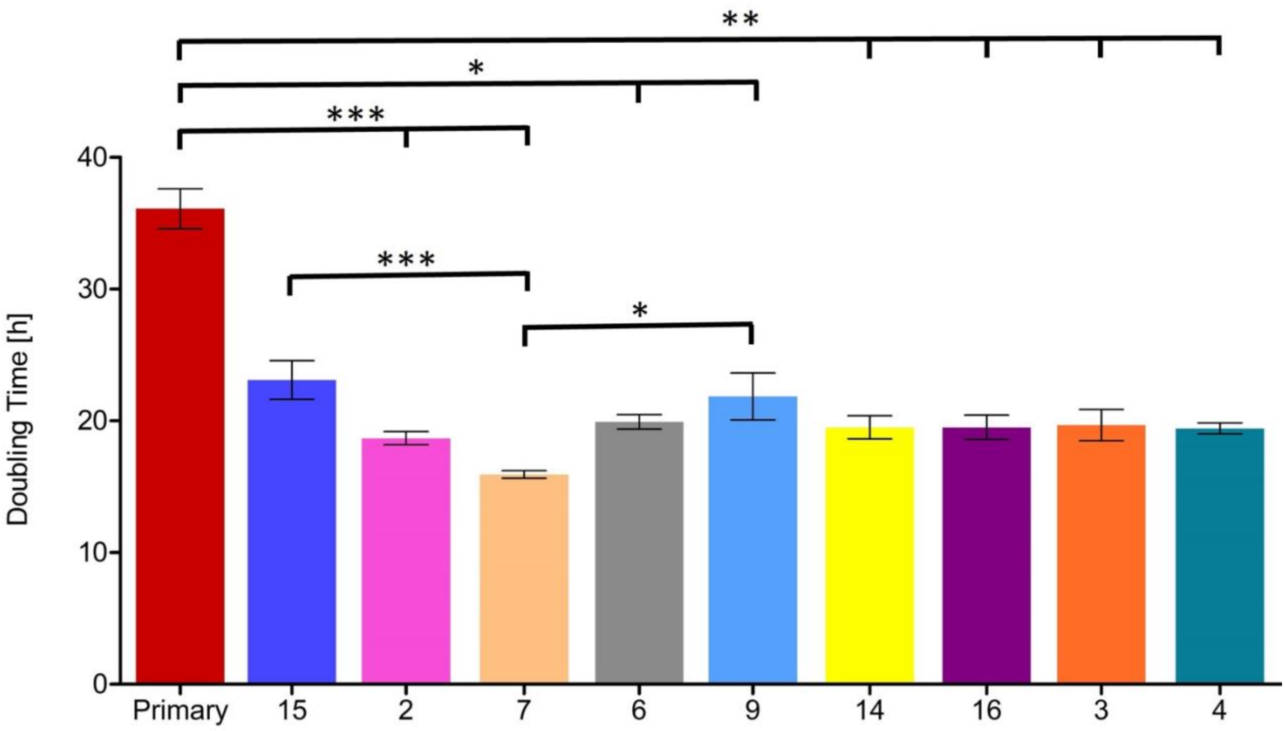

Supplementary Figure 4. Mean DT over four consecutive passages. The mean DT values of primary juvMSC and nine juvMSC clonal cell lines revealed clearer differences in proliferation potential. The primary cells had a significantly higher average DT (36.1 ± 3.3 h). Clones 6 (19.9 ± 1.7 h) and 9 (21.8 ± 4.9 h) showed intermediate values, while clone 15 (23.1 ± 4.0 h) had the highest DT among the clones, making it the closest match to primary cells in terms of proliferation rate (S4). Importantly, no statistically significant differences were found between most of the clones, indicating that their proliferation capacities are broadly comparable with DTs ranging from 18.7 to 19.9 hours. Statistical analysis was performed using the Kruskal–Wallis test. Bars represent mean values from 12 independent measurements per group (n = 12); error bars indicate standard deviations, and statistical significance is denoted as follows: *p < 0.05, **p < 0.01, ***p < 0.001, ***p < 0.0001.

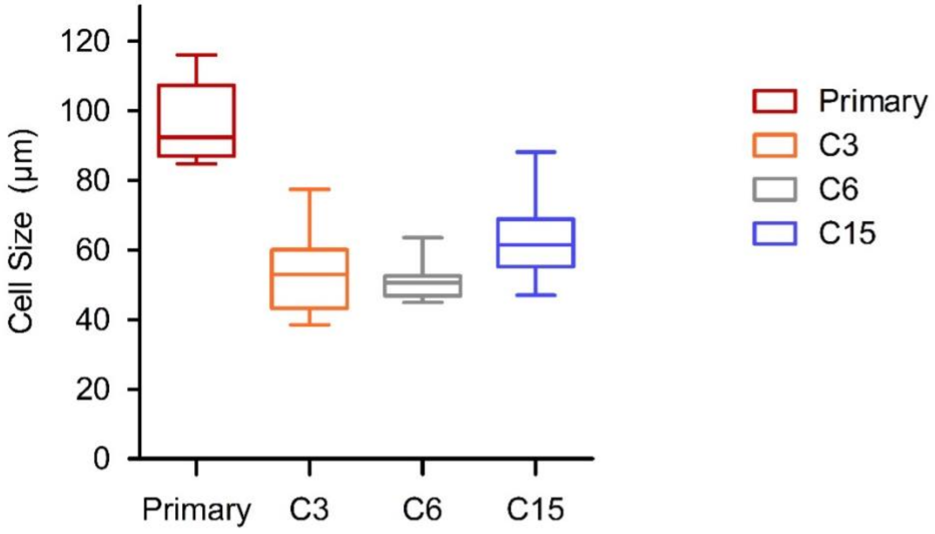

Supplementary Figure 5. Average cell size. The cell size in µm is displayed for primary juvMSC and clones 3, 6, and 15. All clones were significantly smaller than primary cells. Clone 15 (62.89 µm) was the most similar to the primary cells (96.90 µm), followed by clone 3 (53.51 µm) and clone 6 (51.00 µm). Phase contrast microscopic images of primary juvMSC in passage two and of C15 juvMSC in passage 12 were obtained using a Leica DMi1. For cell size quantification, the cells were evaluated using FIJI (ImageJ, NIH, Bethesda, MD, USA). One linear measurement was taken for each cell along the longest and the shortest axes and the measurements were averaged. One-way ANOVA followed by Tukey's Multiple Comparison Test was used for statistical analysis. Data are mean values from 12 cells per group; error bars indicate SD. Statistical significance is denoted as *$p < 0.05$, **$p < 0.01$, ***$p < 0.001$.

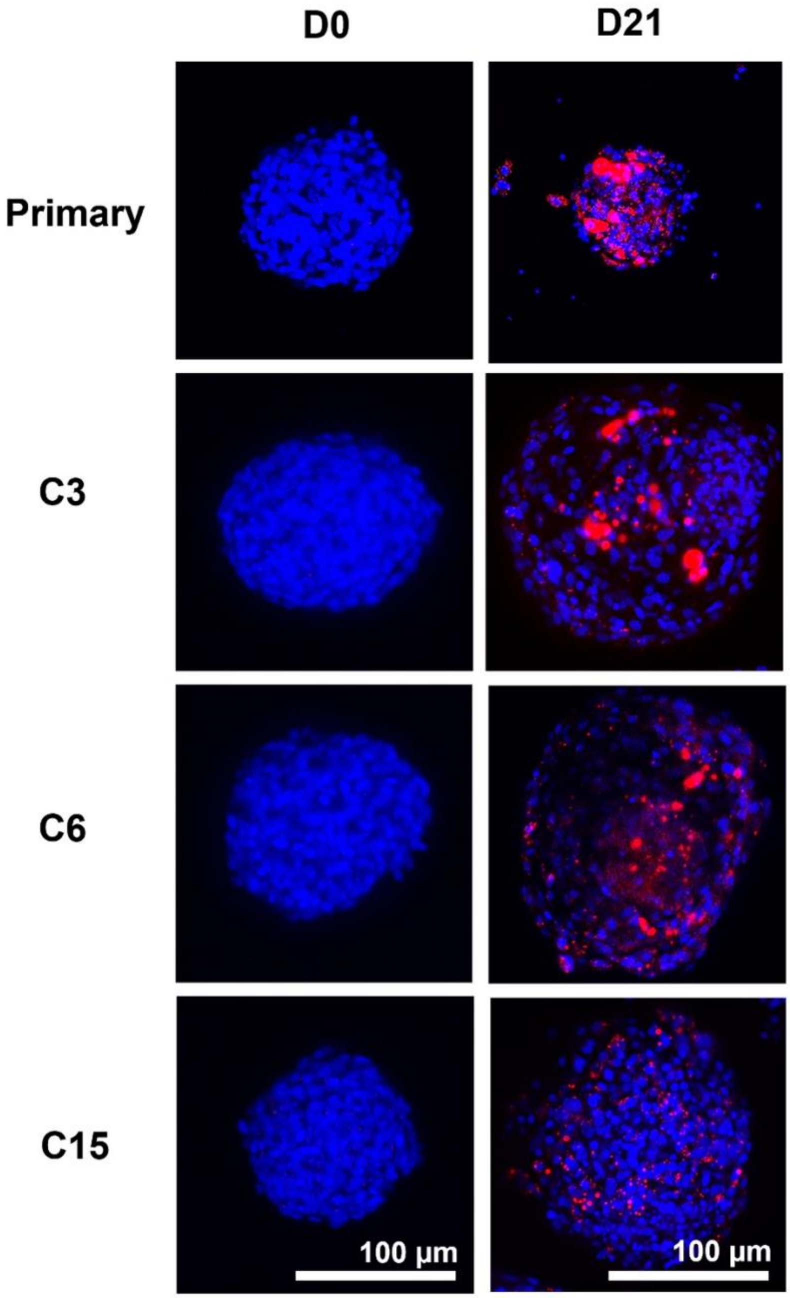

Supplementary Figure 6. Adipogenesis in 3D. Representative images of adipogenic differentiated MSC spheroids after 0 and 21 days of primary juvMSC, clone 3, 6, and 15 are shown. Lipid vacuoles were stained with Nile Red (red), and nuclei were counterstained with DAPI (blue). Staining and imaging was performed as described in Section 2.9.1. After 21 days, primary spheroids exhibited both small and large lipid vacuoles distributed throughout the entire spheroid volume. Clone 3 displayed larger vacuoles concentrated centrally with smaller vacuoles in the periphery, clone 6 demonstrated a uniform distribution of small vacuoles, and clone 15 was characterized by a high number of small, granule-like vacuoles dispersed throughout the spheroid.

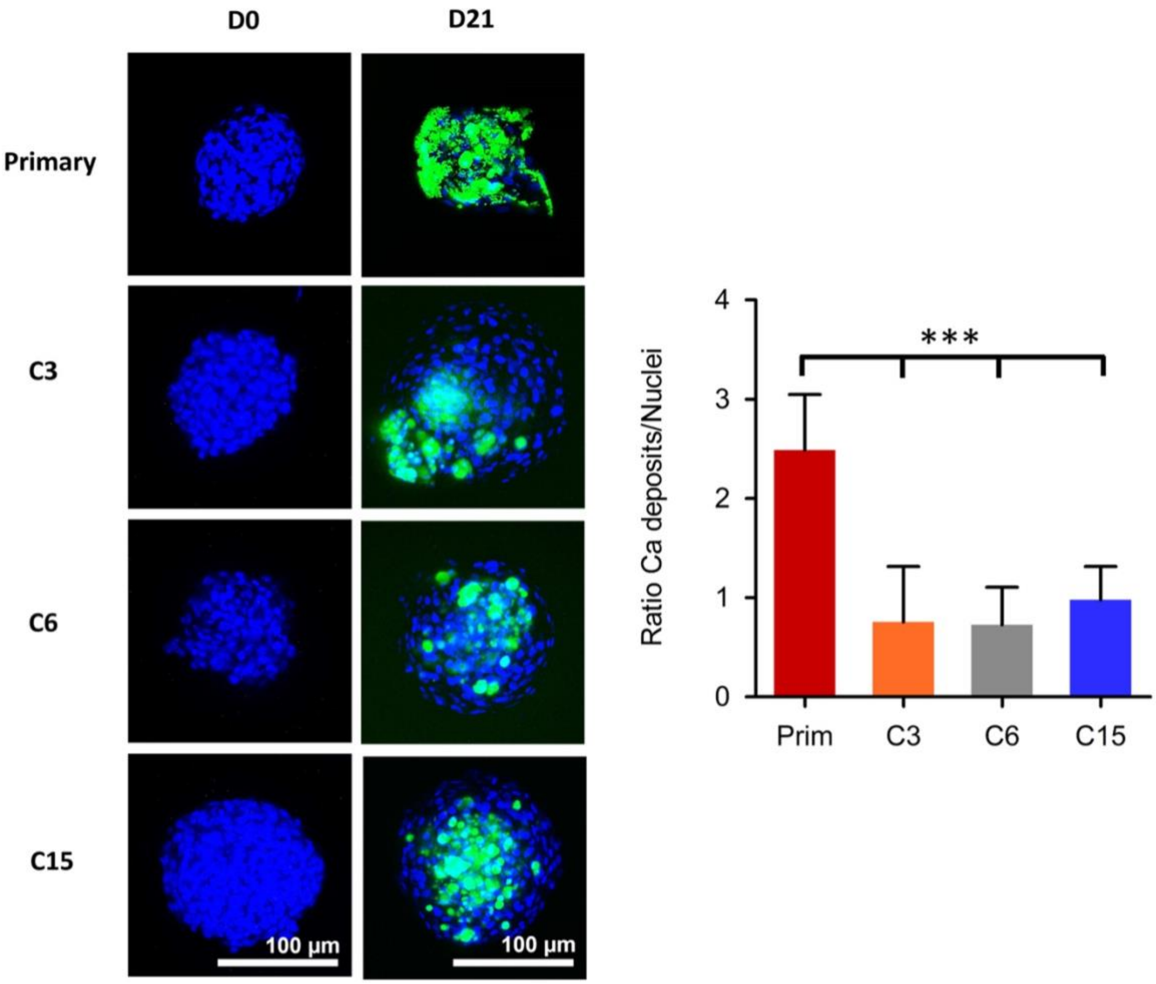

Supplementary Figure 7. Osteogenesis in 3D. Osteogenic differentiated spheroids derived from primary juvMSC, clone 3, 6, and 15 after 21 days were analyzed. (A) Representative images of spheroids after 0 and 21 days of differentiation. Calcium phosphate was stained with Calcein (green), and nuclei were stained with DAPI (blue). Staining and imaging was performed as described in Section 2.9.1. In all three tested clonal lines, larger calcium deposits were predominantly localized to the spheroid core, with smaller deposits extending toward the periphery, and only minimal deposition observed at the spheroid edge. (B) Calcium deposit to nuclei ratio after 21 days of osteogenic differentiation. Measurement was conducted as described in Section 2.9.2. All values represent the mean of five independently imaged and analyzed spheroids from at least three different wells for each cell type and time point, the error bars indicate the standard deviation. Statistical analysis was performed using one-way ANOVA followed by Tukey's Multiple Comparison Test, revealing no significant differences among the clonal lines ($p > 0.05$). Data normality was confirmed using the Shapiro-Wilk test.

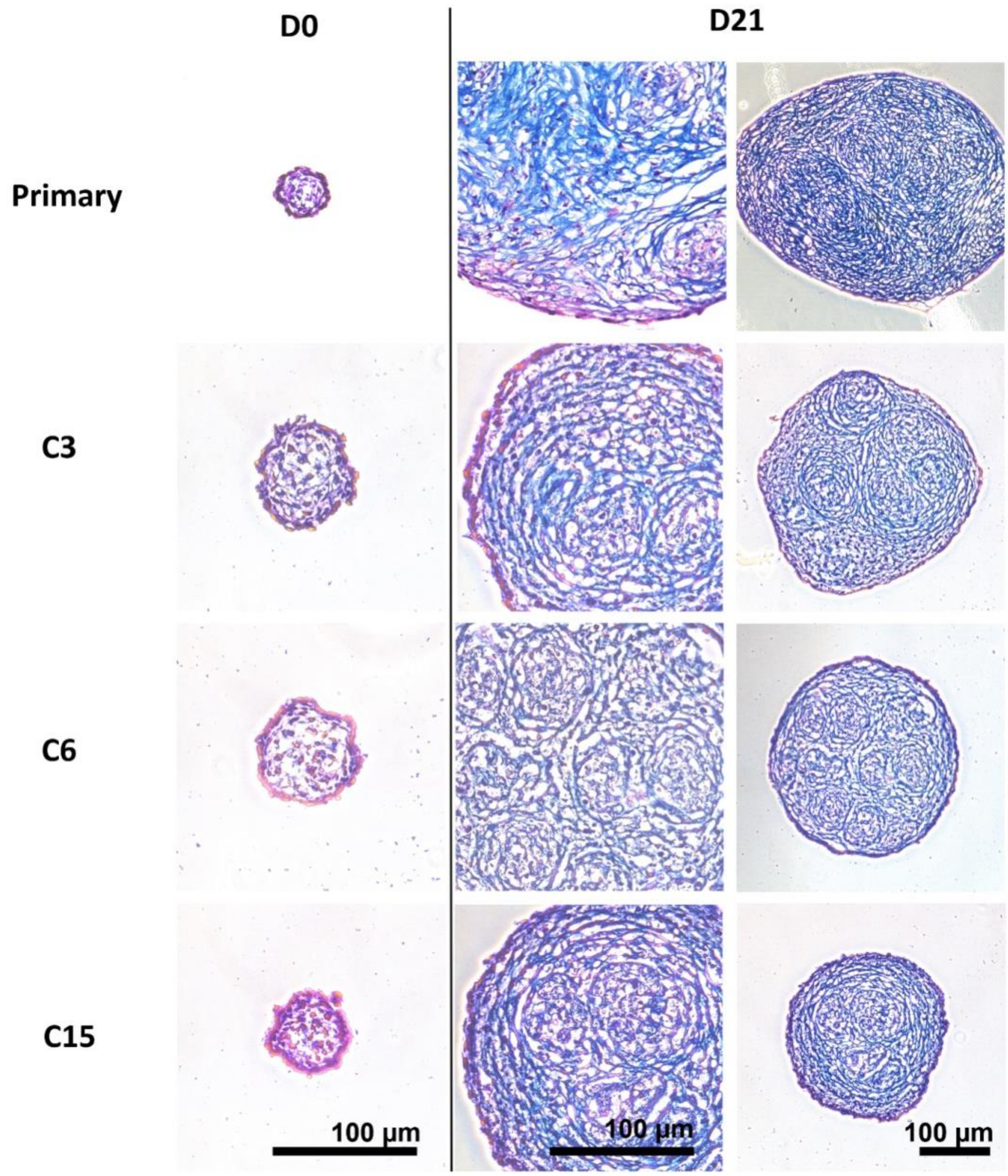

Supplementary Figure 8. Chondrogenesis in 3D. Representative histological images of chondrogenic differentiated MSC spheroids after 0 and 21 days of primary juvMSC, clone 3, 6, and 15 are shown. sGAG in blue were stained with Alcian Blue, while cell nuclei were stained in purple with Nuclear Fast Red. Staining and imaging was performed as described in Section 2.9.1. Following 21 days of chondrogenic induction, all spheroids demonstrated intense Alcian Blue staining, indicative of robust sGAG accumulation.

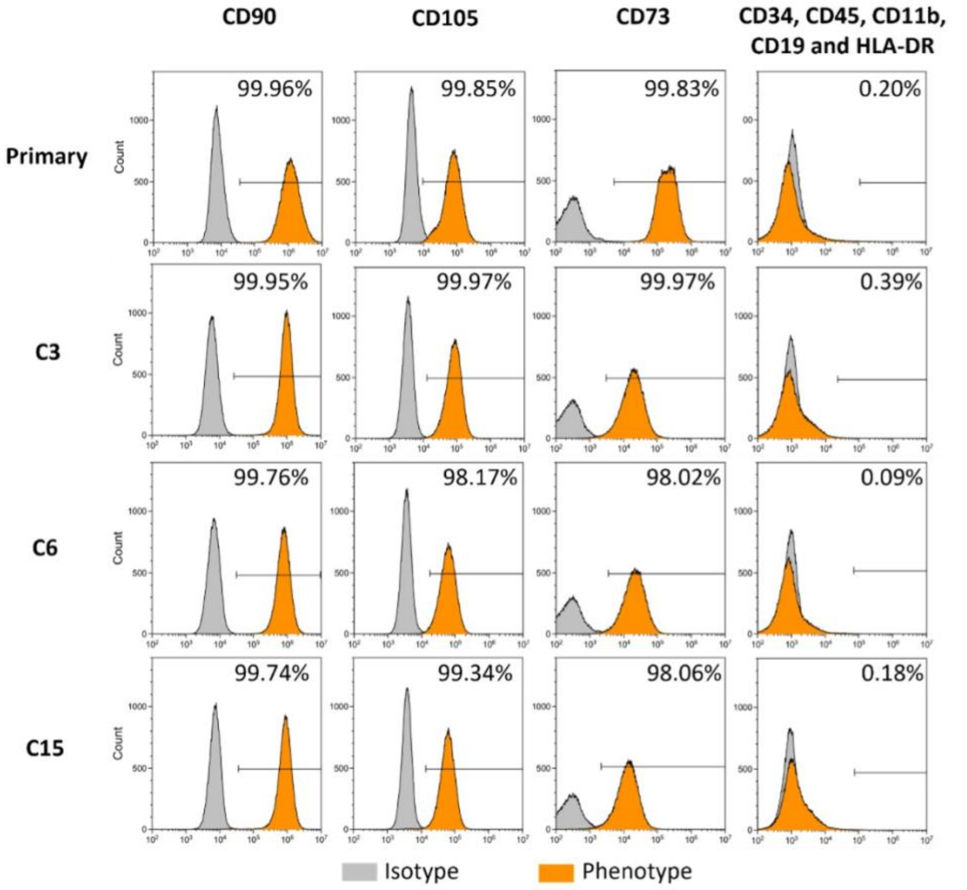

Supplementary Figure 9. Flow cytometric analysis of MSC marker expression. Measurements were performed as described in Section 2.4. Data represents 100,000 acquired events per sample. C3, C6,

and C15 met the ISCT minimal criteria for MSC, with expression of key MSC markers CD90, CD105, and CD73 above 98% and showed ≤0.39% positivity for exclusion markers.

Analysis Report for Cell Line Authentication

1. Sponsor

Ms. Julia Pammer
BOKU Wien
Muthgasse 18
1190 Vienna

2. Analysis Report

Report ID: 02969_018499
Report Version: 01
Issue Date: 21.10.2025
Report approved by: Anna Schmidhofer

3. Descriptions

Customer Test Item ID: juvMSC C15
Analysis Method: Profiling of the human cell lines was done using highly polymorphic short tandem repeat loci (STRs). STR loci were amplified using the PowerPlex® 16 HS System (Promega). Fragment analysis was done on an ABI3730xl (Life Technologies) and the resulting data were analyzed with GeneMarker HID software (Softgenetics).

4. Analysis Results

4.1. Summary Table of the STR Profile

Locus	Chromosomal Location	Core STR Marker	Customer Sample Typed Alleles	Database Alleles	Comments
D3S1358	Chr03		17/18	N/A	
TH01	Chr11	Yes	7/9	N/A	
D21S11	Chr21		30/36	N/A	
D18S51	Chr18		13/15	N/A	
Penta_E	Chr15		11/18	N/A	
D5S818	Chr05	Yes	13	N/A	
D13S317	Chr13	Yes	12	N/A	
D7S820	Chr07	Yes	12/13	N/A	
D16S539	Chr16	Yes	9	N/A	
CSF1PO	Chr05	Yes	11	N/A	
Penta_D	Chr21		9/10	N/A	
AMEL	X/Y	Yes	X/Y	N/A	
vWA	Chr12	Yes	17/18	N/A	
D8S1179	Chr08		10/13	N/A	
TPOX	Chr2	Yes	9/11	N/A	
FGA	Chr04		21/22	N/A	

Microsynth AG
Schützenstrasse 15 · CH-9436 Balgach · Phone +41 71 722 83 33 · info@microsynth.ch · www.microsynth.com

4.2. Electropherogram

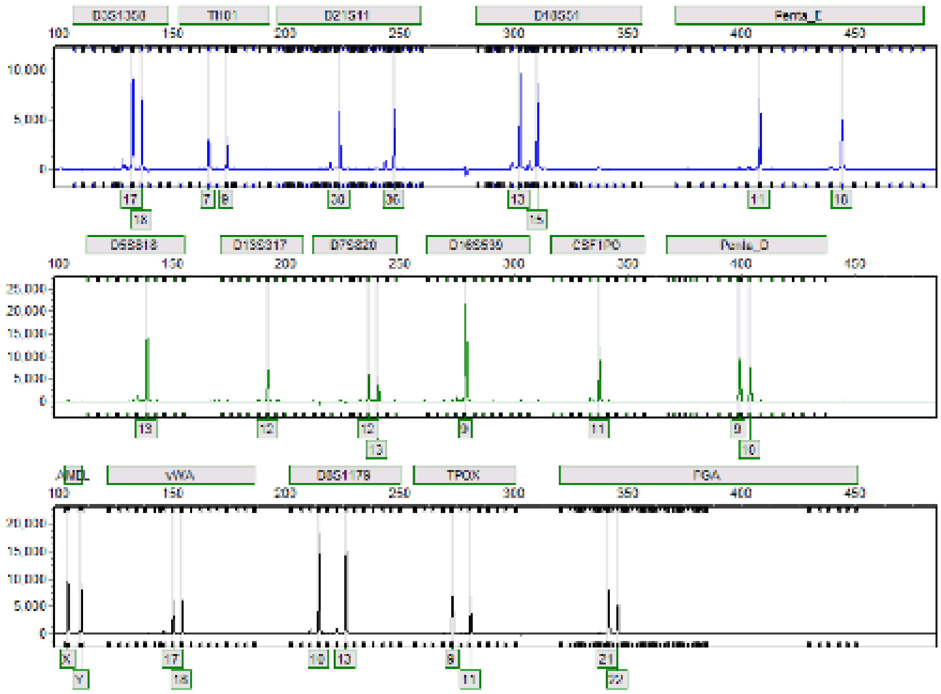

5. Conclusion

According to our analysis of the submitted sample there is no detectable contamination with human origin.

For juvMSC C15 there is no STR reference data available. A search with the analyzed data in the cellosaurus database did not give any useful match with a reference STR profile.

6. Customer Comment

No specific customer comments were provided for this test item

7. Glossary

Short Tandem Repeats (STRs)

Short tandem repeats (STRs) consist of a DNA motif of 2-13 bases that are repeated up to several hundred times. The number of repeats in a STR is highly variable among individuals, resulting in fragment length differences if amplified using PCR. These differences in fragment lengths at different loci are used for profiling the cell lines.

Stutter Peaks

Stutter peaks are small peaks which occur immediately before or after the true peak. Stutter peaks are commonly caused by a slippage of the polymerase during the PCR amplification.

Detection of Cell Line Mixtures

Contamination of one cell line by one or several other cell lines can be detected down to a frequency of the contaminating cell line of 10%. Typically, cell line mixtures will result in STR profiles including three or more peaks for single or multiple loci. If Microsynth notices a possible contamination of a cell line, we will comment the finding in the conclusion part of the analysis.

Peak height ratio

Peak height ratio <25 % (to the highest peak within a STR) is mentioned in the summary table (comments). Peak height ratios <25% need not necessarily have an effect on the behaviour or characteristics of the cell line. A small peak height may be due to reduced amplification efficiency, for example resulting from a mutation in the primer site. The reason for the difference in peak heights observed, however, would need some in depth analysis of the test item.

8. General Comment

The results refer only to the portion of the sample Microsynth has analyzed. The analysis results might not be assigned unconditionally to the whole sample. Microsynth shall not in any event be liable for incidental, consequential or special damages in relation to carried out analyses and corresponding results.

This report is the confidential property of the client addressed. The report may only be reproduced in full. Publication of extracts from this report is not permitted without written approval from Microsynth.

9. Compliance and Quality Assurance Statement

All aspects of this study were in accordance with ISO 9001:2015 standards. All the applied equipment is qualified and calibrated. The applied methods are validated.

Supplementary Figure 10. STR profiling report for C15 juvMSC.

2 Micro-CT analysis

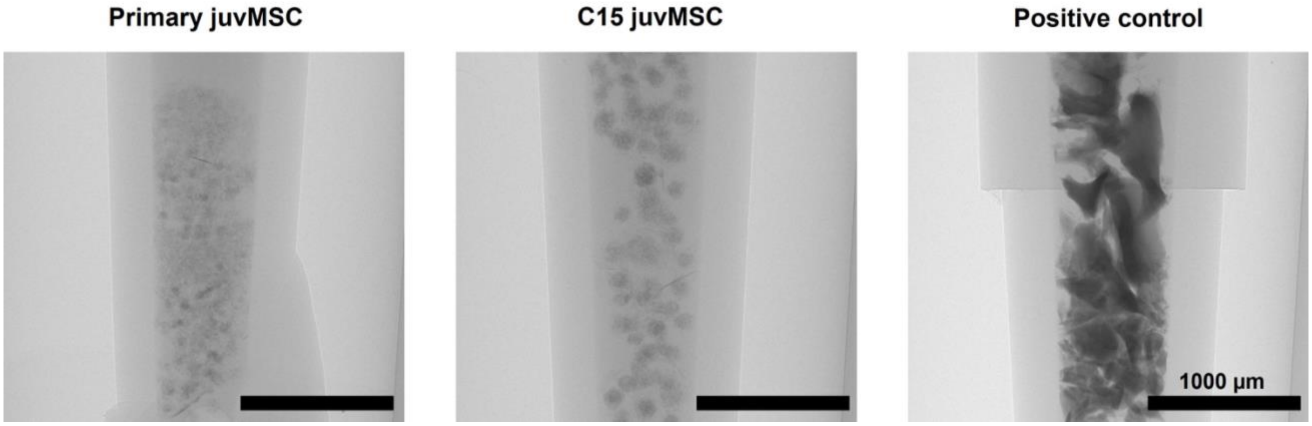

Supplementary Figure 11. High-resolution X-ray radiographs. X-ray radiographs were obtained using a 4X detector assembly. These images depict primary juvMSC and C15 juvMSC spheroids after 21 days of osteogenic differentiation. Decellularized human bone served as positive control.

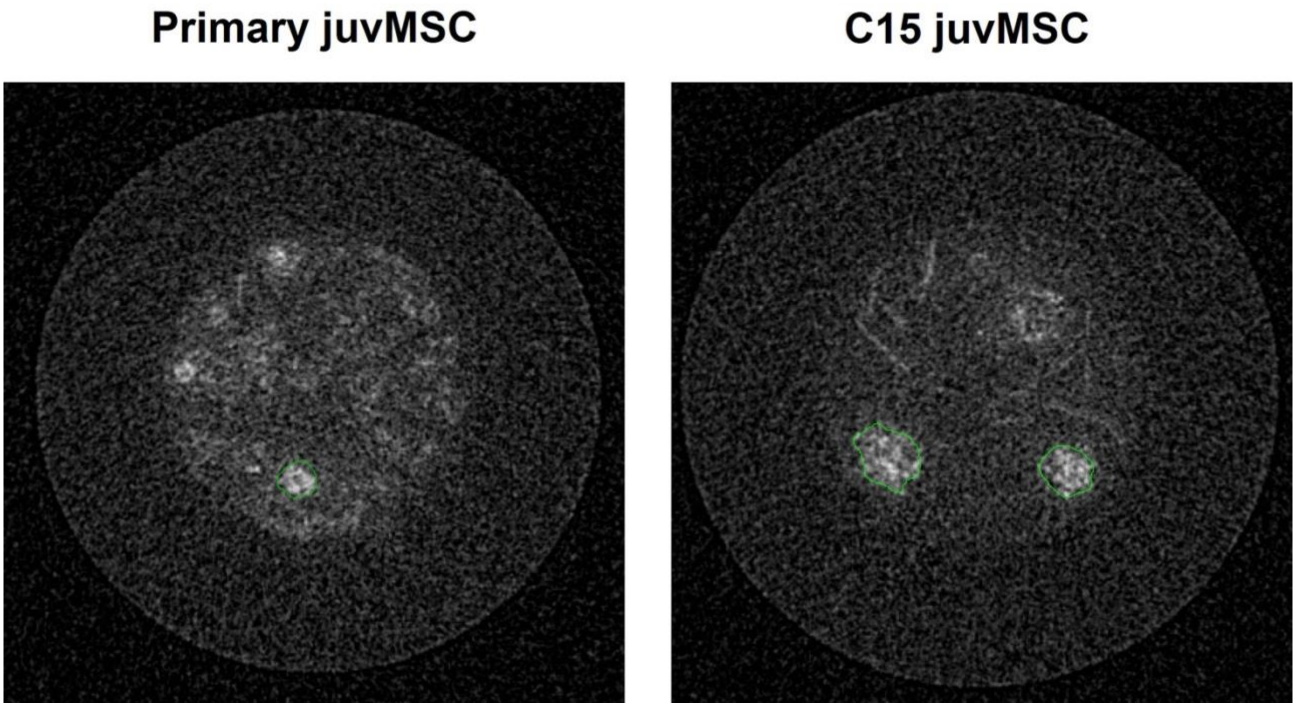

Supplementary Figure 12. Segmentation masks for mineral density measurements. Segmentation mask of the mineralized core of primary and C15 juvMSC spheroids on day 21. Due to the limited contrast and resolution, the periphery of the spheroids could not be included in mineral density measurements.

Videos are attached as separate files. **S13** is depicting primary juvMSC spheroids, **S14** C15 juvMSC spheroids.

Supplementary Videos 13, 14: 3D animation video of primary juvMSC spheroids (S13)/ of C15 juvMSC spheroids (S14). Animation videos based on confocal imaging (Figure 3A) of primary and C15 juvMSC spheroids on day 21 of osteogenic differentiation. Calcium phosphate crystals are visualized in green by Calcein staining. Cell nuclei are stained with DAPI and shown in blue. The showed spheroid section is 30 µm thick.

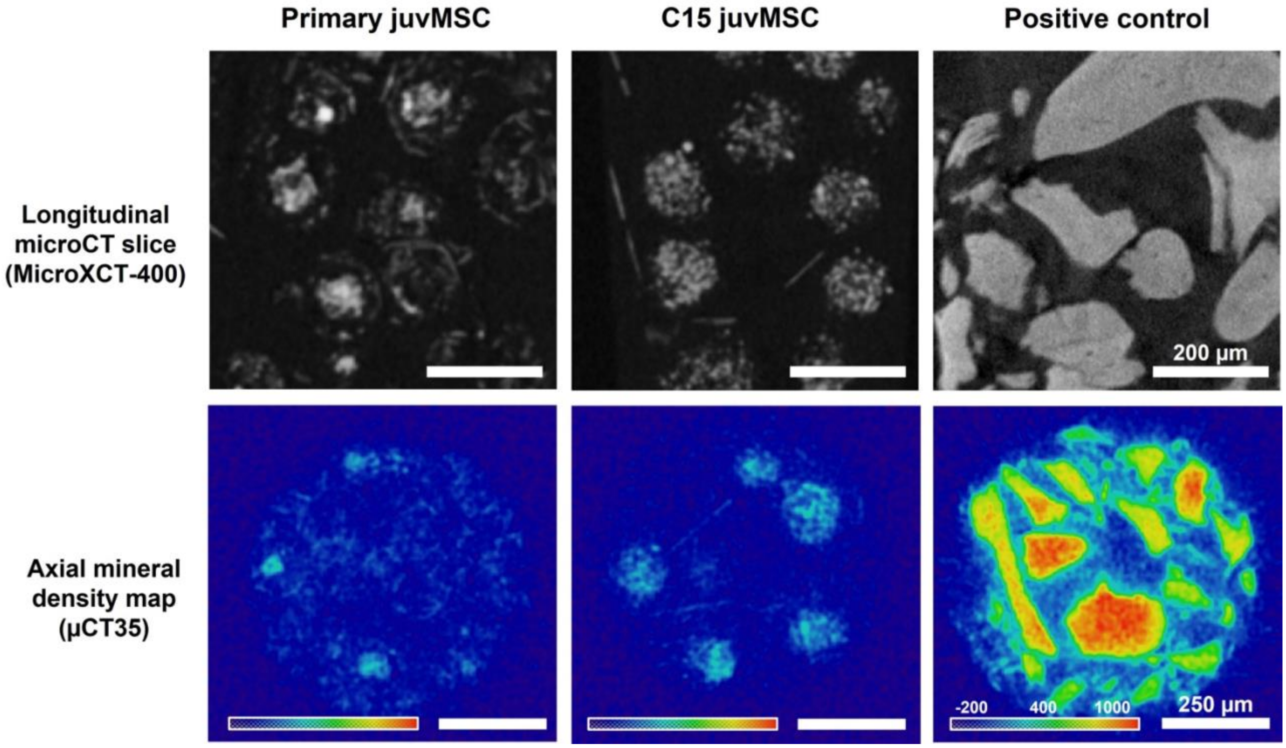

Supplementary Figure 15. Longitudinal microCT slides and axial mineral density maps. Longitudinal microCT slices (XRadia MicroXCT-400) and axial mineral density maps (µCT 35, SCANCO) shown for primary and C15 juvMSC spheroids on day 21 of osteogenic differentiation, and for a positive control. Density calibration in mineral density maps is given in mgHA/cm^3.
